# Cell-Type-Specific Recruitment of Amygdala Interneurons to Hippocampal Theta Rhythm and Noxious Stimuli In Vivo

**DOI:** 10.1016/j.neuron.2012.04.022

**Published:** 2012-06-21

**Authors:** Thomas C.M. Bienvenu, Daniela Busti, Peter J. Magill, Francesco Ferraguti, Marco Capogna

**Affiliations:** 1 Medical Research Council Anatomical Neuropharmacology Unit, Department of Pharmacology, University of Oxford, Mansfield Road, Oxford OX1 3TH, UK; 2 Department of Pharmacology, Innsbruck Medical University, Peter Mayr Str. 1a, A-6020 Innsbruck, Austria

## Abstract

Neuronal synchrony in the basolateral amygdala (BLA) is critical for emotional behavior. Coordinated theta-frequency oscillations between the BLA and the hippocampus and precisely timed integration of salient sensory stimuli in the BLA are involved in fear conditioning. We characterized GABAergic interneuron types of the BLA and determined their contribution to shaping these network activities. Using in vivo recordings in rats combined with the anatomical identification of neurons, we found that the firing of BLA interneurons associated with network activities was cell type specific. The firing of calbindin-positive interneurons targeting dendrites was precisely theta-modulated, but other cell types were heterogeneously modulated, including parvalbumin-positive basket cells. Salient sensory stimuli selectively triggered axo-axonic cells firing and inhibited firing of a disctinct projecting interneuron type. Thus, GABA is released onto BLA principal neurons in a time-, domain-, and sensory-specific manner. These specific synaptic actions likely cooperate to promote amygdalo-hippocampal synchrony involved in emotional memory formation.

## Highlights

► Comprehensive definition of interneuron types of the basolateral amygdala ► Identification of the target subcellular domains of each cell type ► GABAergic cell-type-specific coding of hippocampal theta rhythm and sensory stimuli ► Axo-axonic interneurons are excited by salient sensory stimuli

## Teaser

Bienvenu et al. provide a comprehensive characterization and in vivo recordings of basolateral amygdala interneurons. Their findings suggest that GABA is released onto BLA principal neurons in a time-, subcellular domain-, and sensory-dependent manner.

## Introduction

Understanding how the brain processes emotions holds major potential for fundamental and medical research. Precisely timed neuronal activity across brain regions is crucial for cognitive processing (Singer, 1999). Studies in humans (Richardson et al., 2004) and rodents (Maren and Fanselow, 1995) indicate that cooperation between amygdala and hippocampus is critical for emotional memory formation. This communication involves the synchronization of neuronal activity at theta (θ) frequencies (4–10 Hz) across the basolateral amygdala complex (BLA) and the CA1 hippocampal field. In fear conditioning, a model of emotional memory, animals learn to associate a negative emotional valence to an initially neutral stimulus (e.g., a tone) after its repetitive pairing with an aversive stimulus (e.g., an electrical footshock) (LeDoux, 2000). Unconditioned animals show hippocampus-related θ oscillations in BLA at the levels of individual principal cells and neuron populations (as reflected in local field potentials, LFPs) (Paré and Gaudreau, 1996). Amplitude and power of this rhythm increase after auditory, contextual or social fear learning (Jeon et al., 2010; Paré and Collins, 2000; Seidenbecher et al., 2003). Moreover, the degree of θ synchrony between BLA and CA1 after fear conditioning predicts memory performance (Popa et al., 2010). Precise timing of activity in the BLA is likely important not only for oscillations. It may also be critical for memory encoding, by selectively assigning emotional valence to incoming sensory stimuli. However, how BLA network activities are coordinated remains unknown.

Several lines of evidence suggest that GABAergic neurons may be instrumental in controlling θ oscillations and integrating salient sensory stimuli in the BLA. The BLA is a cortical-like area; in cortex, GABAergic interneurons can synchronize the activity of large cell assemblies (Bonifazi et al., 2009; Cobb et al., 1995). Persistent BLA θ oscillations are accompanied by fear extinction deficits in GAD65 knockout mice (Sangha et al., 2009). Furthermore, electrical footshocks evoke synchronous GABAergic currents in BLA principal neurons (Windels et al., 2010).

GABAergic cells in the BLA are comprised of several groups (McDonald, 1982; Sosulina et al., 2010), with diverse neurochemical expression profiles (Jasnow et al., 2009; Mascagni and McDonald, 2003; Rainnie et al., 2006; Smith et al., 2000). These might play specific physiological roles. However, GABAergic cell types of the BLA have not been fully characterized, and there is a pressing need to define the nature and function of such cellular diversity (Ehrlich et al., 2009). A division of labor between GABAergic cell types in controlling local network activities is exemplified in hippocampus, where cells innervating distinct neuronal compartments fire at specific oscillation phases (Klausberger et al., 2003; Tukker et al., 2007). We hypothesized that BLA GABAergic cells contribute in a type-specific manner to the coordination of θ oscillatory interactions with the hippocampus and local responses to salient sensory stimuli. We investigated this by recording the spontaneous and noxious stimulus-driven firing of anatomically-identified BLA interneurons in vivo. Our findings demonstrate that distinct types of BLA GABAergic cell fulfill specialized and complementary roles in regulating behaviorally relevant network activities.

## Results

We simultaneously recorded spontaneous single-neuron activity in BLA (comprised of the lateral and basal nuclei) and hippocampal θ oscillations in dorsal CA1 (dCA1) LFPs of urethane-anesthetized rats. Prominent θ oscillations (4.15 ± 0.23 Hz, mean ± SD) occurred during cortical activated states in dCA1 (Klausberger et al., 2003), but not in BLA LFPs. Gamma (γ) oscillations were also detected in dCA1 LFPs (42.1 ± 1.60 Hz, mean ± s.d.).

We recorded interneuron responses to noxious stimuli by delivering electrical shocks and pinches to the hindpaw controlateral to the recording sites. We also examined the firing of BLA glutamatergic principal neurons in relation to dCA1 θ. After recordings, neurons were juxtacellularly filled with Neurobiotin, allowing for their unambiguous identification.

Interneurons with somata in the BLA were recorded and labeled (Figure S1, available online, shows cell locations). These were GABAergic, as all tested cells expressed the vesicular GABA transporter (VGAT) and/or glutamate decarboxylase (GAD; Figures 3F and 4I), and all synapses examined with electron microscopy were symmetric. Interneuron types were distinguished according to the combination of their postsynaptic targets, neurochemical markers and axo-dendritic patterns. Twenty eight GABAergic cells could be classified in four types: axo-axonic, parvalbumin-expressing basket, calbindin-expressing dendrite-targeting, and “AStria-projecting” cells.

### Axo-Axonic Cells Increase Their Firing in Response to Noxious Stimuli

**Figure 1:**
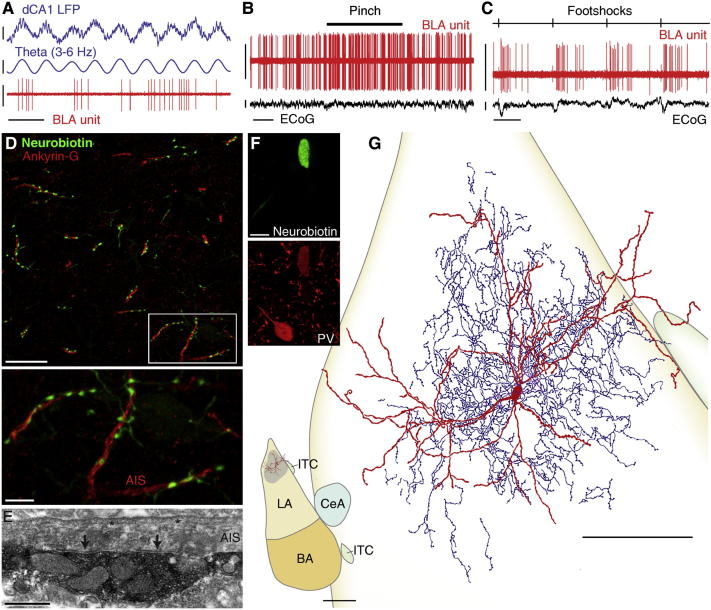
Axo-axonic Cells: Firing In Vivo and Anatomical Characterization (A) In vivo, the neuron tjx20f fired non θ-modulated spike trains. (B) tjx20f increased its firing rate in response to hindpaw pinches. The electrocorticogram (ECoG) shows stable global activation. (C) Another representative axo-axonic cell (tjx56b), dramatically increased firing upon hindpaw electrical shocks. (D) Close appositions between tjx63a axon varicosities and ankyrin G-expressing axon initial segments (AIS). Low (top) and high magnification (of area delineated, bottom) of projection of a confocal z stack of 4.42 μm thickness. (E) Electron micrograph of a DAB-labeled axon bouton (tjx20f) forming two synaptic junctions (arrows) with a single AIS. ^∗^undercoating. (F) tjx20f is immunopositive for parvalbumin (PV; single confocal optical section). (G) Reconstruction of tjx20f. Soma and entire dendritic tree (red) are drawn from 5 sections of 60 μm thickness. Axon (blue, main axon is purple) is drawn from 2 sections, for clarity. Inset: position of tjx20f dendrites (in red) in the BLA and axonal field extent (gray area) estimated from the two drawn and surrounding sections. Boundary colors apply to the main panel. LA: lateral amygdala, BA: basal amygdala, CeA: central amygdala, ITC: intercalated cells cluster. Orientation: top: dorsal, right: medial. Scale bars: (A) LFP raw and filtered: 0.4 mV, unit: 1 mV, time: 400 ms; (B) unit: 1 mV, ECoG: 0.25 mV, time: 4 s; (C) unit: 1 mV, ECoG: 0.25 mV, time: 1 s; (D) top: 20 μm, bottom: 5 μm; (E) 500 nm; (F) 10 μm; (G) 100 μm, inset 500 μm. See also Figures S1–S5 and Tables S1–S3.

Axo-axonic cells (n = 6) were recorded and anatomically indentified. During dCA1 θ, they spontaneously fired action potentials at a mean frequency of 12.4 Hz (range 6.5–15.9 Hz; Table 1; Figure 1A). The firing of 4 of 6 cells was significantly modulated in phase with dCA1 θ (p < 0.005, Rayleigh test), albeit weakly (mean modulation depth (r) = 0.05, see Experimental Procedures). Two cells fired independently from the hippocampal θ rhythm (Figure 1A). The four θ-modulated cells fired preferentially between the peak and the descending phase of dCA1 θ (range 187.0–283.7°, where 0° and 360° represent θ troughs; θ phase histograms of single neurons are illustrated in Figure S2). However, statistical analysis showed that these four cells did not form a synchronized population in relation to dCA1 θ (R′ = 1.03, R_0.05,4_ = 1.09, Moore test). Furthermore, the firing of axo-axonic cells did not show statistically significant modulation in phase with dCA1 γ oscillations (p > 0.1, Rayleigh test, n = 6; Figure S3; Table S3).

Axo-axonic cells displayed dramatic short-latency excitations in response to noxious stimuli. All axo-axonic cells increased their firing rates upon hindpaw pinches (+377% of baseline, latency 267 ms, peak 377 ms, n = 6; ranges: 133%–606%, latency 200–400 ms, peak 400–600 ms, respectively; Table 2; individual histograms are shown in Figure S4). This excitation rapidly adapted, and was curtailed at stimulus offset (Figure 5D). Responses to electrical footshocks were similarly pronounced (mean 226% of baseline, latency 50 ms, peak 225 ms, n = 4/4; ranges 133%–606%, 20–100 ms, 20–420 ms, respectively; Figure 1C; Table 2; individual histograms, Figure S5).

These neurons exhibited typical axo-dendritic patterns. Their axons formed cartridges. Almost all of large-axon varicosities were in close apposition with ankyrin G-expressing axon initial segments, (n = 6/6 cells), as seen with immunofluorescence (Figure 1D). We analyzed randomly-sampled synapses from two of these cells using electron microscopy. The vast majority of postsynaptic targets were axon initial segments (95.4%, n = 43 synapses; Figure 1E; Table S1), confirming that these cells were of the axo-axonic type. All axo-axonic cells expressed parvalbumin (PV), sometimes weakly (Figure 1F), but were never calbindin (CB)-positive. Two of 6 neurons densely expressed the GABA_A_R-α1 subunit on their dendrites (immunohistochemical results are summarized in Table S2). Axo-axonic cells were bitufted. Their dendrites did not branch immediately, were tortuous and sparsely spiny (Figure 1G). Axonal arborizations of all 6 cells were very dense and mostly contained within the dendritic field. Axons were always restricted to the BLA, but could be distributed between lateral and basal nuclei.

These results show that the firing of axo-axonic cells of the BLA dramatically increases in response to salient sensory stimuli. However, their spontaneous population activity is not tightly synchronized with hippocampal θ (Figure 5).

### Parvalbumin-Expressing Basket Cell Assemblies Tonically Inhibit Principal Cells

**Figure 2:**
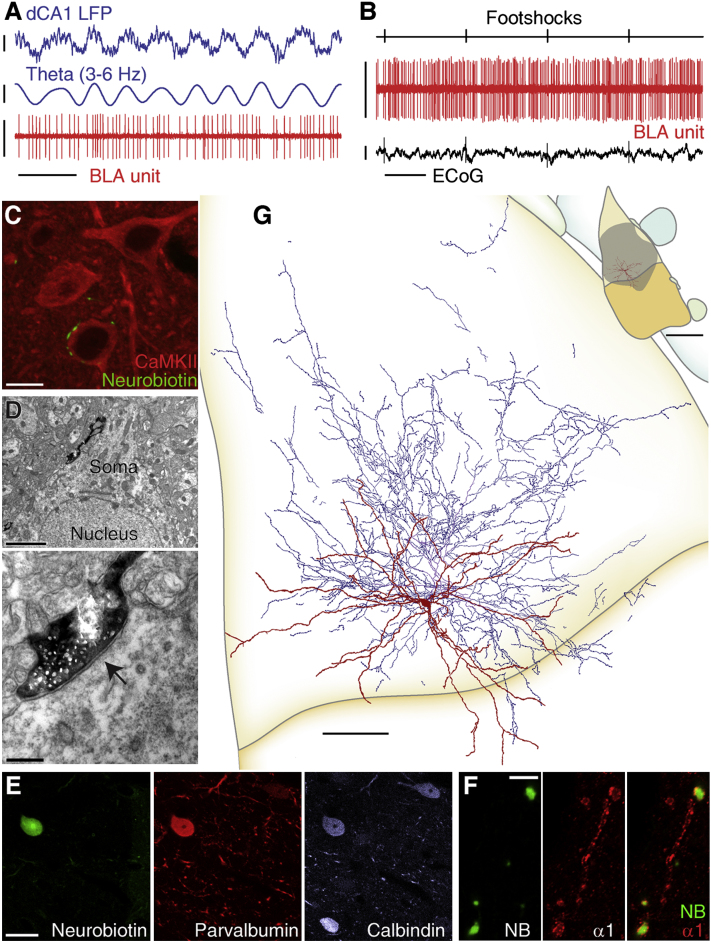
PV Basket Cells: Firing In Vivo and Anatomical Characterization All panels show data from the same neuron (tjx48a). (A) Tonic, weakly modulated firing during hippocampal θ oscillations. (B) Moderate firing increases in response to hindpaw electrical shocks, not noticeable in the raw data. (C) Axon varicosities making close appositions with the soma of a CaMKIIα^+^ principal neuron (projection of a confocal z stack of 3.29 μm thickness). (D) Low (top) and high magnification (bottom) electron micrographs of a synapse (arrow) made by a labeled axon bouton with a soma. (E) Immunopositivity for parvalbumin and calbindin. Immunofluorescence confocal images (single optical section). (F) Accumulation of GABA_A_R-α1 (α1) at the dendritic membrane of the Neurobiotin (NB)-filled cell (single confocal optical sections). (G) Reconstruction of tjx48a. Soma and entire dendritic tree are drawn from 12 sections of 60 μm thickness. Axon is drawn from 2 sections for clarity. Inset: position of tjx48a dendrites in the BLA and axonal field extent estimated from the two drawn and surrounding sections (color code as in Figure 1G). Orientation: top: dorsal, right: medial. Scale bars: (A) LFP raw and filtered: 0.4 mV, unit: 1 mV, time: 400 ms; (B) unit: 1 mV, ECoG: 0.25 mV, time: 1 s; (C) 10 μm; (D) top 2 μm, bottom 250 nm; (E) 20 μm; (F) 5 μm; (G) 100 μm, inset 500 μm. See also Figures S1–S7 and Tables S1–S3.

Next, we studied the firing of parvalbumin-expressing (PV^+^) basket cells (n = 15). During dCA1 θ oscillations, PV^+^ basket cells fired at a mean frequency of 11.0 Hz (range 1.8–27.2 Hz; Table 1), some tonically (coefficient of variation (CV) < 1, n = 8/15), others irregularly (CV > 1, n = 7/15). It has been frequently speculated that PV^+^ basket cells pace θ rhythms in the BLA (reviewed in Ehrlich et al., 2009). Instead, we found that most cells were only weakly modulated with dCA1 θ (mean r = 0.06; Figure 2A), and at dispersed phases (Table 1; Figures 5B and S2). In keeping with this, the firing of PV^+^ basket cells as a population was not synchronized with this rhythm (R' = 0.73, R_0.05,12_ = 1.042, Moore test; Figure 5A). The firing of PV^+^ basket cells was not modulated with dCA1 γ oscillations (p > 0.04, Rayleigh test, n = 15; Figure S3; Table S3).

As with θ modulation, PV^+^ basket cells displayed heterogeneous and generally moderate responses to noxious stimuli (Figure 2B; Table 2). Half of the cells tested (6/12) were excited by hindpaw pinches, three were inhibited, two showed an excitation-inhibition sequence, and one cell did not respond significantly (Figure S4). Several cells tested (5/11) were inhibited by electrical footshocks, three cells were excited, and three other cells did not change their firing rates (Figure S5). Cells that were excited in response to one type of noxious stimulus could be inhibited by the other stimulus (Table 2). This further shows that the firing of PV^+^ basket cells is not selectively tuned by noxious stimuli. Importantly, heterogeneous firing among PV^+^ basket cells does not reflect spatial segregation of activity patterns in the BLA (see Figure S1A and Table 1).

Axon varicosities of these cells were large and clustered. Light microscopic analysis (n = 12 cells) revealed that they mostly made close appositions with somata and large dendrites of BLA neurons expressing the calcium/calmodulin-dependent kinase II alpha subunit (CaMKIIα; Figure 2C), a marker of principal cells (Supplemental Experimental Procedures). Electron microscopic analysis confirmed that the main postsynaptic targets were somata (55%; n = 40 synapses, 2 cells; Figures 2D and S6C) and proximal dendrites (45%; diameter 1.29 ± 0.1 μm; Figures S6A and S6B; Table S1). For 72.5% of these synapses, the postsynaptic target was unambiguously identified as a CaMKIIα+ principal neuron (Figures S6A and S6C, Table S1). Thus, our results established that these interneurons were basket cells.

In addition to PV, these cells always expressed CB and an accumulation of the GABA_A_R-α1 subunit along their somatodendritic plasma membranes (n = 12/12 cells; Figures 2E and 2F; Table S2). This neurochemical pattern is distinct from those of the other cell types studied here. Three PV^+^ neurons were classified as basket cells based on these features, although their axons could not be analyzed. In addition, PV^+^ basket cells displayed characteristic axonal and dendritic fields. They were multipolar. Their dendrites were varicose, typically aspiny, straight, and branched rarely (Figure 2G). Axonal arborizations were dense within the dendritic field and extended beyond it in radial branches, sometimes over long ranges (Figure S7A). This suggests that some PV^+^ basket cells influence neuronal activities in large parts of the BLA. Overall, PV^+^ basket cells show distinct postsynaptic targets and neurochemical contents, demonstrating they are different cell types in the BLA.

As a group, PV^+^ basket cells do not appear to fire tuned to dCA1 θ or noxious stimuli (Figure 5). Thus, assemblies of them may tonically inhibit principal neurons. The finding that axo-axonic and PV^+^ basket cell groups do not fire in synchrony with hippocampal θ rhythm raises the question of which interneurons might fulfill this role.

### Calbindin-Expressing Dendrite-Targeting Cells Fire Synchronously with Hippocampal Theta Oscillations

**Figure 3:**
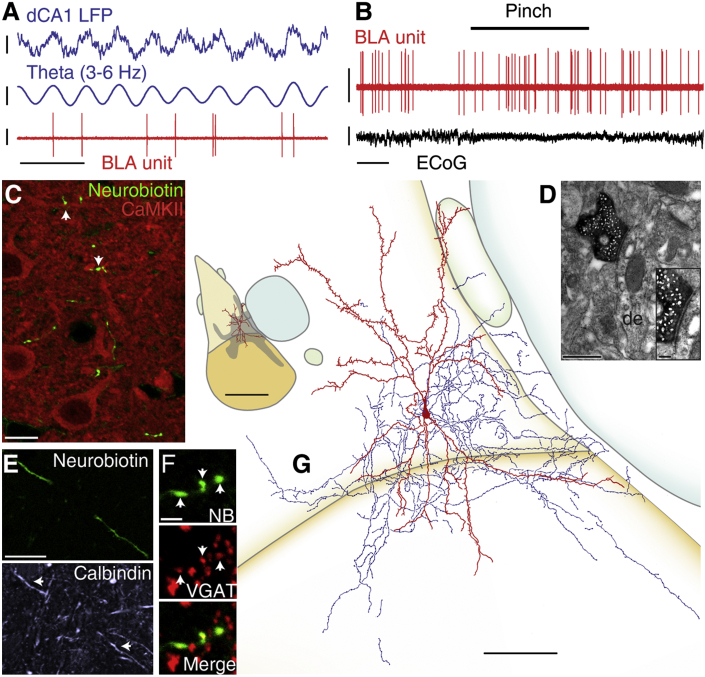
CB^+^ Dendrite-Targeting Cells: Firing In Vivo and Anatomical Characterization (A) tjx22c fired preferentially before the peak of dCA1 θ. (B) tjx59b did not change its firing rate during a noxious stimulus. (C) Axon varicosities of tjx21i avoid CaMKII^+^ somata and occasionally make visible appositions with CaMKII^+^ dendrites (arrows; projection of a confocal z stack of 2.10 μm thickness). (D) Electron micrograph of a synapse formed by an axon bouton of tjx22c with a CaMKIIα^+^ dendrite (de). Inset: higher magnification. (E) tjx22c is immunopositive for calbindin (structured illumination, single optical section). (F) tjx22c is GABAergic: confocal images (projection of z stack of 0.75 μm thickness) showing Neurobiotin (NB)-filled axon varicosities containing VGAT (arrows). (G) Reconstruction of tjx22c. Soma and entire dendritic tree are drawn from 8 sections of 60 μm thickness. Axon is drawn from 2 sections, for clarity. Inset: position of tjx22c dendrites in the BLA and axonal field extent estimated from the two drawn and surrounding sections (color code as in Figure 1G). Orientation: top: dorsal, right: medial. Scale bars: (A) LFP raw and filtered: 0.4 mV, unit: 1 mV, time: 400 ms; (B) unit: 1 mV, ECoG: 0.5 mV; (C) 10 μm; (D) 500 nm, inset 100 nm; (E) 20 μm; (F) 2 μm; (G) 100 μm, inset 500 μm. See also Figures S1–S6 and Tables S1–S3.

Dendrite-targeting CB^+^ cells spontaneously fired at a mean frequency of 3.5 Hz (range 3.0–4.3 Hz, n = 3; Table 1). Their firing was consistently and strongly modulated with the late ascending phase of dCA1 θ (Figure 3A; mean angle 144.9°, mean r = 0.13; Figures 5B and S2; Table 1). Thus, as a population, CB^+^ dendrite-targeting cells did fire tightly synchronized with hippocampal θ (R′ = 1.15, R_0.05,3_ = 1.095, p < 0.05, Moore test; Figure 5A). In contrast, none of these cells fired in phase with dCA1 γ (p > 0.1, Rayleigh test, n = 3; Figure S3; Table S3).

Responses to hindpaw pinches could be tested in two cells. One cell did not significantly change its firing (Figure 3B); the other was inhibited (latency 4.2 s, peak 4.4 s; Table 2; Figure S4). Electrical footshocks were applied during recording of the third cell. In this experiment, only 53 shocks were applied and no change in firing was observed. Such a sample size is a limitation of the juxtacellular recording/labeling technique used. It cannot be ruled out that more heterogeneous activity relationships with θ oscillations or sensory stimuli would emerge if a larger sample of CB^+^ cells were available.

When examined with light microscopy, axons of the three cells were distributed in the BLA neuropil. Some axon varicosities made close appositions with dendrites of CaMKIIα^+^, principal neurons. A substantial proportion was not in apposition with identifiable CaMKIIα^+^ structures (Figure 3C) and likely contacted small dendritic processes that could not be resolved with light microscopy. Electron microscopic analysis demonstrated that postsynaptic targets were exclusively dendrites of small to medium diameter (0.59 ± 0.05 μm, n = 41 synapses, 2 cells; Figure 3D; Table S1). Notably, this diameter value was the smallest among the neuron types studied (p < 0.05, Kruskal-Wallis test with Dunn's multiple comparison; Figure S6E). In 24% of these synapses, targets were confirmed to be CaMKIIα^+^ dendrites of principal neurons (Figure 3D).

In addition to strongly expressing CB (Figure 3E), two neurons tested contained very low levels of PV in their somata (but no detectable PV in their dendrites). One cell was GABA_A_R-α1^+^. The cells were immunonegative for other molecules tested, including somatostatin (Table S2). Dendrites emerged in bipolar arrangement from the soma. They were tortuous, rough, and sometimes spiny. Axons and dendrites were restricted to the BLA, but could span lateral and basal nuclei (Figure 3G).

These results show that CB^+^ dendrite-targeting cells represent a specific cell type, whose firing is synchronized with CA1 θ (Figure 5A).

### Amygdalo-striatal Transition Area-Projecting Neurons Are Inhibited by Noxious Stimuli

**Figure 4:**
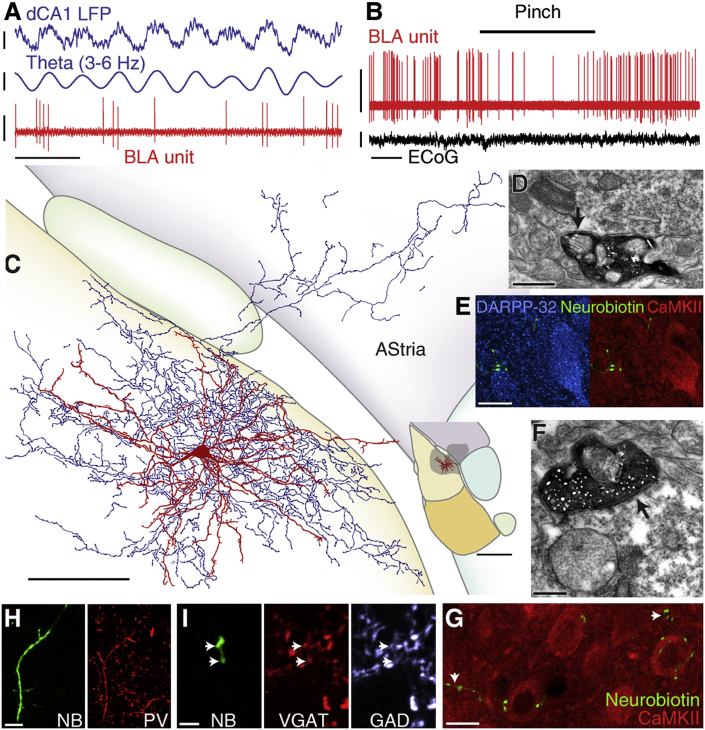
AStria-Projecting Cells: Firing In Vivo and Anatomical Characterization All panels show data from one neuron (tjx52a). (A) Preferential firing during the ascending phase of hippocampal θ oscillations. (B) Reduced firing during hindpaw pinches. (C) Reconstruction of tjx52a. Soma and entire dendritic tree are drawn from 5 sections of 60 μm thickness. Axon is drawn from 2 sections, for clarity. Inset: position of tjx52a dendrites in the BLA and axonal field extent in the two drawn and surrounding sections. Color code is as in Figure 1G, and AStria is purple. Orientation: top: dorsal, right: medial. (D) Electron micrograph of a labeled bouton making a synapse (arrow) with a CaMKIIα^+^ soma in the AStria. (E) Axon varicosities making close appositions with a principal neuron (CaMKIIα^+^/DARPP-32^+^) soma in the AStria. Projection of a confocal z stack of 3.06 μm thickness. (F) Electron micrograph of a labeled bouton making a synapse (arrow) with a CaMKIIα^+^ principal cell dendrite in the lateral amygdala. (G) Axon varicosities making close appositions with principal neuron somata (CaMKIIα^+^) or unidentified structures (arrows) in the lateral amygdala (projection of confocal z stacks of 8.05 μm thickness). (H) Immunopositivity for parvalbumin (structured illumination z stack; NB: Neurobiotin). (I) tjx52a is GABAergic: confocal images (projections of z stack of 0.94 μm thickness) showing axon varicosities (arrows) positive for VGAT and GAD. NB: Neurobiotin. Scale bars: (A) units and LFPs: 0.5 mV, time: 400 ms; (B) units 1 mV, time: 4 s; (C) 100 μm, inset 500 μm; (D) 500 nm; (E) 10 μm; (F) 250 nm; (G) 10 μm; (H) 10 μm; (I) Scale bar: 2.5 μm. See also Figures S1–S7 and Tables S1–S3.

**Figure 5:**
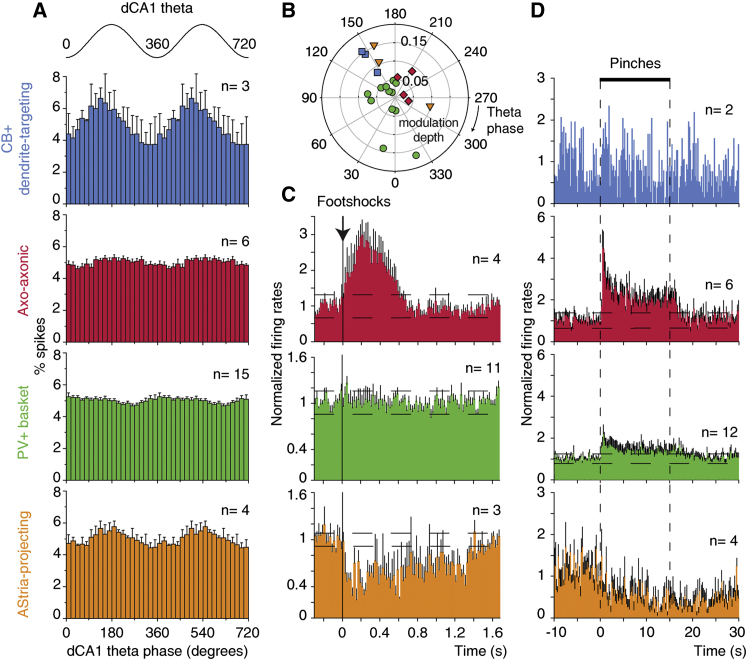
Firing Modulation with Hippocampal Theta Oscillations and Responses to Noxious Stimuli Are Cell Type Specific (A) Cell types mean θ phase histograms. CB^+^ dendrite-targeting interneurons are homogeneously and strongly modulated in phase with dCA1 θ, in contrast to the other cell types. Two θ cycles are represented for clarity. 0°, 360°, and 720°: θ troughs. (B) Polar distribution of individual neurons' preferred θ phases and modulation depths. Each symbol represents a significantly modulated cell. (C and D) Cell types mean peristimulus histograms for noxious stimulation. Axo-axonic cells are dramatically excited and AStria-projecting cells are inhibited by electrical shocks and pinches delivered to the controlateral hindpaw. Error bars: SEM; dashed lines: mean ± 2 standard deviations. n values represent the number of neurons tested in each analysis. See also Figures S2, S4, and S5.

We discovered a GABAergic cell type that projects to the amygdalo-striatal transition area (AStria, hence its name), as well as innervating the BLA (Figures 4C and S7B). The firing of most AStria-projecting cells (mean frequency 4.01 Hz, range 3.4–6.0 Hz, n = 4; Table 1) was related to dCA1 θ (n = 3/4, mean r = 0.12). Two of these cells preferentially fired before the peak (Figure 4A) and one fired most during the descending phase of the θ rhythm (Figures 5B and S2; Table 1). As a result, this cell population was not statistically phase-locked to hippocampal θ (R′ = 0.86, R_0.05,3_ = 1.095, Moore test). The firing of AStria-projecting neurons was not modulated with dCA1 γ oscillations (p > 0.04, Rayleigh test, n = 4; Figure S3; Table S3).

In contrast to the previous three cell types, AStria-projecting cells were robustly inhibited by noxious stimuli. Hindpaw pinches suppressed the firing of 3/4 cells tested (Figure 4B; mean latency 2,133 ms, peak 2,200 ms; ranges, 1,000–3,800 ms for peak and latency; Table 2; Figure S4). In two cells, this inhibition persisted for several seconds after the pinch offset (Figure 5D). Electrical footshocks also elicited strong inhibitory responses in AStria-projecting cells (−85% of baseline, latency 33 ms, peak 380 ms, n = 3; ranges: 75%–100%, 20–60 ms, 20–740 ms, respectively; Figures S5 and 5C).

The axon projecting to the AStria innervated somata and dendrites of DARPP-32^+^ cells, likely medium-sized spiny neurons (Anderson and Reiner, 1991), which also expressed CaMKIIα (Figures 4D, 4E, and S6D). Most of the axons were distributed in the BLA, where they made dense ramifications (Figures 4C and S7B). Studied with light microscopy, a proportion of the large axon varicosities made multiple perisomatic contacts with CaMKIIα^+^ BLA principal neurons; the others possibly contacted small dendrites (Figure 4G). Electron microscopic analysis confirmed that postsynaptic targets in the lateral nucleus were dendrites (Figure 4F) and somata (35% and 65%, respectively, n = 40 synapses, 2 cells; Table S1). Of these, 35% were confirmed CaMKIIα^+^ neurons (Figure 4F, Table S1). Dendrites targeted by AStria-projecting neurons were smaller than those postsynaptic to PV^+^ basket cells but larger than those targeted by CB^+^ dendrite-targeting cells (diameter 0.79 ± 0.06 μm, p < 0.05; Figure S6E).

All AStria-projecting neurons expressed PV (Figure 4H), and half also expressed CB. GABA_A_R-α1 was moderately enriched in the plasma membrane of one cell but was never strongly expressed, in contrast to PV^+^ basket cells (Table S2). Dendrites were multipolar and branched profusely. They were short, smooth, and very tortuous (Figures 4C and S7B).

The distinct dendritic and axonal patterns and postsynaptic targets demonstrate that AStria-projecting cells may constitute a specific cell type. The present data indicate that they do not form a synchronous cell population with respect to dCA1 θ but dramatically decrease their firing in response to noxious stimuli (Figure 5).

Overall, various BLA interneuron types appear to fire differently in relation to network activities. However, they could not be separated on the basis of their spike shapes and durations (Figure S8; Table S5).

### BLA Principal Neurons Fire Heterogeneously in Relation to Hippocampal Theta Oscillations

Next, we assessed the firing modulation of glutamatergic principal neurons in phase with hippocampal θ, because they are a major target of the interneurons defined above and represent the main output of the BLA (n = 23 cells; see Figure S1B for somata locations). Principal cells fired at very low rates during hippocampal θ (mean 0.29 Hz, range: 0.03–1.34 Hz; n = 23; Table S4). Irregular burst firing (2–3 spikes) was often observed, as reflected in high coefficients of variation of firing (CVs, which quantify irregularity of spike trains, 1.95 ± 0.13). Noteworthy, we found that principal cells fired longer-lasting spikes than all four types of interneurons (Figure S8; Table S5). Unsupervised cluster analysis could differentiate principal cells and interneurons (Figure S8C).

**Figure 6:**
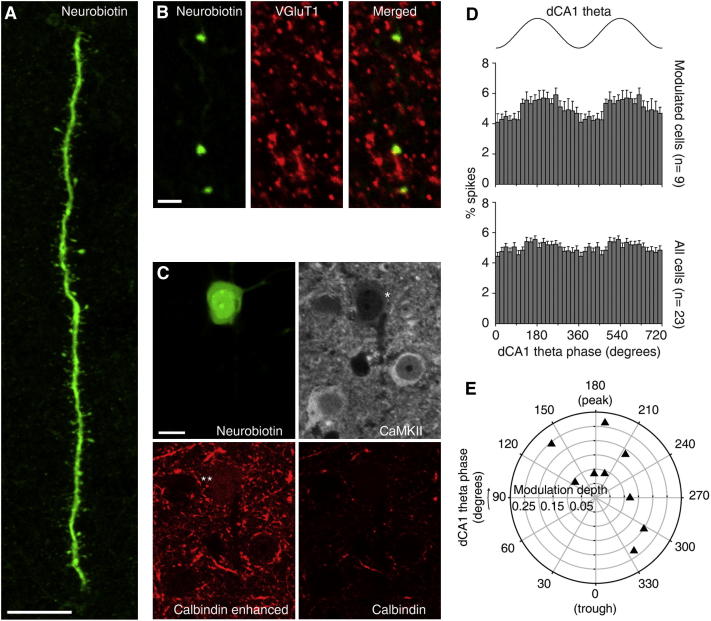
Anatomical Features and Theta Modulation of BLA Principal Neurons (A) The neuron tjx89c had large dendrites covered with spines (projection of a confocal z stack of 7.99 μm thickness). (B) tjx89c expressed VGluT1, demonstrating its identity as a glutamatergic neuron (single optical section confocal micrographs; recordings from this neuron are shown in Figure 7A). (C) Moderate levels of CaMKIIα, (^∗^) as well as weak calbindin (^∗∗^) expression were detected in the soma of tjx89c (confocal images, single optical sections). (D) Phase histograms. Two θ cycles are shown for clarity. Error bars represent SEM. (E) Polar distribution of preferred θ phase and modulation depth of the θ-modulated principal neurons (n = 9/23). Each symbol represents a modulated neuron. Note that all 9 cells were strongly modulated. Scale bars (A) 10 μm; (B) 2 μm (C) 10 μm. See also Figures S1 and S8, and Table S4.

We verified the identity of 15 recorded neurons after labeling. They showed large dendrites covered with spines (Figure 6A), typical of principal neurons (Faber et al., 2001; McDonald, 1982). All were identified as glutamatergic by the expression of the vesicular glutamate transporter 1 (Figure 6B). They coexpressed CaMKIIα (n = 14/14 tested; Figure 6C; Table S4). Of the remaining eight neurons, three were weakly Neurobiotin-filled cells expressing CaMKIIα, whereas the other five were unlabeled (see Supplemental Experimental Procedures).

The firing of 39% (9/23) of principal neurons was strongly modulated in phase with dCA1 θ oscillations (mean r = 0.17; Figure 6D; Table S4). The majority of BLA principal neurons thus fired independently of dCA1 θ. Theta-modulated cells did not form a tightly synchronized group (R′ = 0.72, R_0.05,9 =_ 1.053, Moore test; Figure 6D), in line with the weak ensemble (LFP) θ activity observed in the BLA. Importantly, the proportion of θ-modulated neurons and the preferred phase distribution (Figure 6E) were both consistent with previous studies in nonanesthetized animals (Paré and Gaudreau, 1996; Popa et al., 2010).

### Modulation with Ventral Hippocampal Theta

The BLA receives dense innervation from the ventral hippocampal formation (McDonald, 1998; Pitkänen et al., 2000), but not from dCA1. However, dCA1 θ oscillations represent a more reliable reference signal compared with ventral hippocampal θ. In dCA1, the θ rhythm is regular, reproducible across animals and it has been suggested to indirectly but accurately reflect ventral hippocampal activities (Royer et al., 2010). Indeed, θ oscillations recorded from dorsal and ventral CA1 are coherent in both urethane-anesthetized and drug-free rats (Adhikari et al., 2010; Hartwich et al., 2009; Royer et al., 2010), and many ventral hippocampal neurons fire phase-locked to dCA1 θ (Hartwich et al., 2009; Royer et al., 2010). In contrast, LFP θ in ventral hippocampus would have been an unsuitable reference. LFP θ phase in ventral hippocampus varies dramatically between recordings, preventing a reliable comparison of phase locking between animals (Hartwich et al., 2009; Table S6). Moreover, ventral hippocampal θ oscillations have low amplitude and occur only transiently (Adhikari et al., 2010; Hartwich et al., 2009; Royer et al., 2010), compromising the isolation of θ epochs using unbiased methods (Csicsvari et al., 1999; Klausberger et al., 2003) and the calculation of θ phases.

To validate that dCA1 signal predicted spike timing of BLA neurons relative to ventral hippocampal θ, we performed experiments that included a vCA1-subiculum electrode (n = 3 animals, 6 neurons). Ventral stratum radiatum LFP signal was used as second reference. Theta oscillations were intermittent and had generally low amplitude, as reported in behaving rodents (Figure S9; Adhikari et al., 2010; Royer et al., 2010).

**Figure 7:**
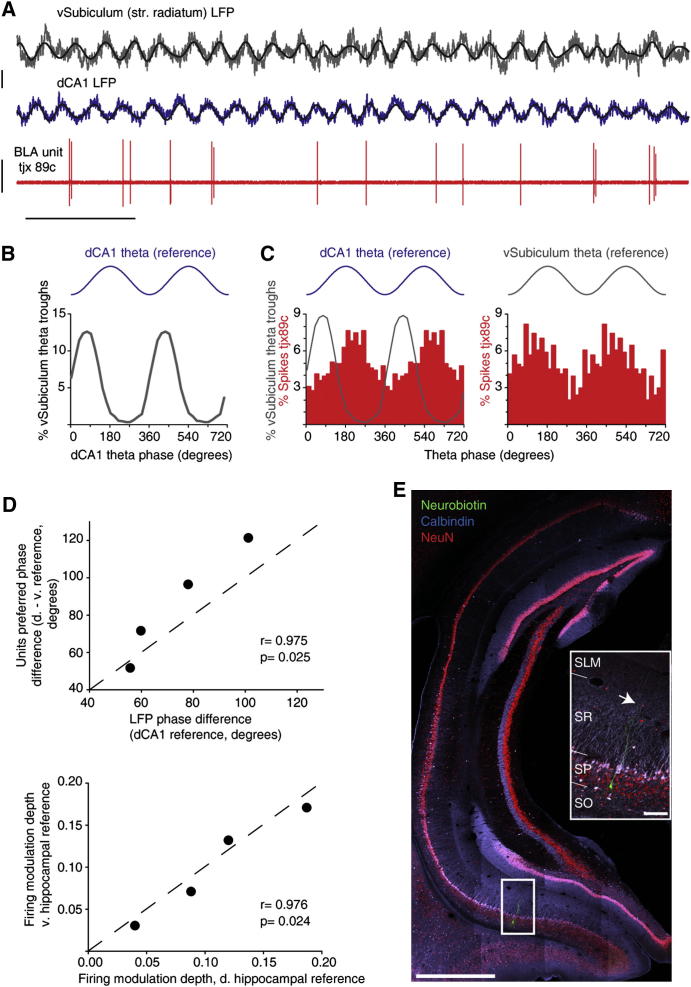
Dorsal CA1 Reference Recapitulates BLA Unit Firing Modulation by Ventral Hippocampal θ (A and B) A principal neuron (tjx89c) was recorded along with LFPs from dorsal (CA1 str. pyramidale) and vHippocampus (subiculum str. radiatum). (A) Raw data showing tjx89c firing during an exemplary period of ventral hippocampal θ. Traces filtered for θ frequencies are overlaid. (B) Theta oscillations in the ventral subiculum are phase-locked to those in dCA1. Phase histogram representing the distribution of vSubiculum θ troughs across the dCA1 θ cycle. (C and D) Single units modulated in phase with dCA1 θ are similarly modulated with vHippocampus θ. (C) Left: phase histogram of tjx89c spikes computed with the dCA1 reference. vSubiculum θ trough distribution was superimposed to illustrate the predicted firing phase relationship of tjx89c with vSubiculum θ rhythm (predicted phase: 135.7°). Right: distribution of tjx89c spike angles relative to vSubiculum θ, consistent with the prediction. (D) Results obtained with dCA1 θ phase as a reference accurately reflect firing modulation of neurons in phase with ventral hippocampal θ. Top: linear correlation between the predicted (LFPs) and actual phase differences of neuron modulation by dorsal and ventral hippocampal θ. Bottom: linear correlation between modulation depths calculated with the dorsal and ventral hippocampal references. Diagonal dashed lines: unity line. (E) Anatomically confirmed recording sites in the ventral subiculum from the same experiment. The white box highlights the Neurobiotin (green) deposit made at the str. pyramidale recording site. NeuN immunoreactivity (red) was used as a panneuronal nuclear and cytoplasmic marker to delineate str. pyramidale. Calbindin immunoreactivity (light blue) highlights hippocampal layers. Colocalization with NeuN is indicated by white and pink. Inset: higher magnification of the area delineated in the main panel. Arrow: recording site in str. radiatum. SO: str. oriens; SP: str. pyramidale: SR, str. radiatum; SLM: str. lacunosum moleculare. Scale bars: (A) time: 1 s, LFPs: 0.5 mV, units: 2 mV; (E) 1 mm, inset: 100 μm. See also Figure S9 and Table S6.

As expected, dCA1 signal predicted BLA unit firing modulation with ventral hippocampal θ. Differences between the phases of dCA1 and vCA1-subiculum LFP θ oscillations were similar to, and correlated with the difference between the preferred phases of neuron firing calculated with the two references (Pearson's correlation r = 0.975, p = 0.025 and circular-circular correlation: Fisher and Lee's method, Oriana software, p < 0.05, n = 4: 3 principal cells, 1 PV+ basket cell; Figures 7 and S9). Moreover, θ modulation strengths of units calculated with dorsal and ventral hippocampal references were similar and linearly correlated (Pearson's correlation r = 0.976, p = 0.024; n = 4; Figure 7D). These results establish that dCA1 is a suitable and sensitive reference to study the coupling of BLA neuron firing to hippocampal θ.

## Discussion

This study defines several types of BLA interneurons and their role in shaping BLA activity in relation to dCA1 θ oscillations and noxious stimuli, two processes critical in forming emotional memories. The key findings are the following: dendrite-targeting CB^+^ interneurons provide inhibition to BLA principal cells in phase with hippocampal θ oscillations. The firing of PV^+^ basket cells is not tightly synchronized with θ oscillations. Axo-axonic cells consistently and dramatically increase their firing in response to noxious stimuli. In addition, we discovered a GABAergic cell type well placed to coordinate spontaneous and sensory-related BLA-AStria interactions. Our results support the hypothesis that interneurons are critical in regulating timing in the BLA, and that they operate in a cell-type-specific manner. We demonstrate that this principle is not limited to firing relationships with ongoing oscillations, but also applies to the integration of sensory information.

### GABAergic Cell Types of the BLA

Defining cell types requires the correlated analysis of molecular markers, full dendritic and axonal patterns and postsynaptic targets at ultrastructural level (Somogyi, 2010). The present study unambiguously defines four interneuron types of the BLA.

First, we demonstrate that axo-axonic and PV^+^ basket cells are two distinct cell types in the rat BLA. Indeed, PV^+^ basket cells target somata and dendrites of principal neurons, whereas axo-axonic cells innervate almost exclusively axon initial segments. Thus, the hypothesis that axo-axonic and PV^+^ basket cells of BLA are a single cell type (Woodruff et al., 2006) should be rejected, at least in adult rats. The present report of an extensive coexpression of PV, CB, and/or GABA_A_R-α1 in BLA interneurons is consistent with earlier studies (McDonald and Betette, 2001; McDonald and Mascagni, 2004). Our data suggest that the coexpression of moderate to high levels of PV, CB, and GABA_A_-Rα1 may be specific to basket cells.

Second, we identified a CB^+^ dendrite-targeting cell type. The existence in the BLA of such PV^+^ interneurons specifically targeting dendrites has been inferred (Muller et al., 2006; Woodruff et al., 2006; Woodruff and Sah, 2007), but never directly demonstrated. The target selectivity of basket and dendrite-targeting cells demonstrates a clear separation, and precludes their grouping into a single population.

Third, we report a specific GABAergic cell type, that we named AStria-projecting, for its axon reaching outside the BLA.

The BLA most likely comprises additional GABAergic cell types (Ehrlich et al., 2009). Indeed, Golgi staining has revealed BLA interneurons with axo-dendritic patterns distinct from those presented here (e.g., neurogliaform-like cells, McDonald, 1982). Moreover, populations of BLA GABAergic neurons lacking PV have been shown to express markers such as calretinin, cholecystokinin, neuropeptide Y, or somatostatin (Spampanato et al., 2011). Recent in vitro studies have elucidated the firing characteristics, dendritic and axonal patterns, expression of neurochemical markers, and functional connectivity of some of these neurons (Jasnow et al., 2009; Rainnie et al., 2006; Sosulina et al., 2010). However, the lack of a comprehensive anatomical strategy has so far prevented a clear characterization of these interneuron types.

We demonstrated that different BLA interneuron types make GABAergic synapses with specific domains of principal cells. This appears of key significance in light of their distinct firing activities.

### Firing Relationship with Hippocampal Oscillations

The firing relation of BLA interneurons to hippocampal θ differed between cell types. This is consistent with only a subset of putative BLA interneurons firing in phase with hippocampal θ in behaving cats (Paré and Gaudreau, 1996). Importantly, the modulation strength of interneuron activity was independent from the power and frequency of dCA1 θ oscillations (Experimental Procedures).

Dendrite-targeting CB^+^ cells showed the most consistent firing modulation. The dendritic inhibition they provide could modulate the integration of glutamatergic inputs and limit action potential back-propagation, thereby rendering synaptic plasticity onto principal neurons dependent on hippocampal θ. This is particularly important in the BLA, where synaptic plasticity on dendritic spines is thought to underlie fear memory encoding (Humeau et al., 2005; Ostroff et al., 2010).

We found weak and inconsistent θ-modulation of PV^+^ basket and axo-axonic cell firing, which both innervate the perisomatic domain of target cells. At the population level, these cells appear to provide constant perisomatic inhibition of principal neurons. We cannot rule out that synchronization is limited to subpopulations of these neurons. Somata of BLA principal cells are innervated by ∼60 PV^+^ boutons and their axon initial segment by ∼20 boutons (Muller et al., 2006). Terminals of PV^+^ fast-spiking cells release GABA with high fidelity (Hefft and Jonas, 2005). Together with our results, this suggests that ∼900 boutons release GABA around each BLA principal cell soma every second. Such powerful inhibition likely contributes to the very low firing rates of principal neurons, provided axo-axonic cells chiefly inhibit postsynaptic cells (Woodruff et al., 2011). Our finding of weakly θ-related activity of perisomatic-innervating cells constitutes a major difference from what has been reported in neocortex and hippocampus (Hartwich et al., 2009; Klausberger et al., 2003). Individual AStria-projecting cells might provide θ-modulated perisomatic inhibition to their target neurons in BLA and AStria, but they do not seem to play such a role as a population.

Interneurons might adjust their relationship with θ rhythms on a fine time-scale, possibly depending on behavioral states. The present analysis assumes relatively stationary activities and was not designed to capture specific bouts of dynamic synchronization. The juxtacellular method used here restricts sample sizes. It is possible that large assemblies of interneurons whose activity is weakly synchronized can still have a large net effect on principal neuron populations.

None of the recorded interneurons showed modulation in phase with dCA1 γ oscillations. This held true for the analysis of θ-nested γ oscillations and for entire γ oscillation periods. Our findings are consistent with γ oscillations being generated locally and indicate that BLA interneurons are more likely to participate in amygdalo-hippocampal synchrony at θ frequencies.

The firing of ∼40% of principal cells was strongly modulated in phase with hippocampal θ. Modulated cells could correspond to the so-called fear neurons, which selectively receive inputs from ventral hippocampus (Herry et al., 2008). As found in behaving rats, preferred θ phases of principal cells were dispersed (Popa et al., 2010). Phase-modulation heterogeneity may result from the convergence at heterogeneous phases of perisomatic inhibition (as our data suggest) and of excitatory inputs from several brain regions. For example, perirhinal and entorhinal cortices also innervate the BLA (McDonald, 1998; Pitkänen et al., 2000) and contain neuronal assemblies oscillating at θ frequencies (Collins et al., 1999).

### Firing Responses to Noxious Stimuli

Salient sensory events recruit the amygdala to attach emotional significance to coincident neutral stimuli (LeDoux, 2000). Previous work suggests that phasic GABAergic inhibition may be instrumental in integrating noxious stimuli, by increasing synchrony in the BLA (Crane et al., 2009; Windels et al., 2010). Diversity in roles played by interneuron types could be expected not only during spontaneous activity, but also in integrating salient sensory stimuli. Indeed, we found cell-type-dependent responses to noxious stimuli.

AStria-projecting neurons responded with a long-lasting inhibition of firing. Their target neurons in amygdala and AStria should be concomitantly disinhibited, perhaps promoting Hebbian synaptic plasticity. While the functions of AStria neurons are unknown, they might be involved in appetitive behavior and potentially participate in a parallel circuit controlling emotional responses.

In contrast, the firing of axo-axonic cells increased systematically and dramatically upon noxious stimuli presentation. Inputs from extrinsic afferents might mediate this effect. The responses of axo-axonic cells to noxious events may trigger the stimulus-induced GABAergic currents recorded in principal cells, thus generating synchrony in the BLA (Windels et al., 2010). Axo-axonic cells could provide temporal precision to large principal cell assemblies for the encoding of associations with unconditioned stimuli, in two ways: (1) by synchronizing principal neurons for glutamatergic inputs subsequently reaching the BLA; (2) by limiting the synaptic integration time window (Pouille and Scanziani, 2001), thus controlling spike-timing-dependent plasticity (Humeau et al., 2005). Activation of GABA_B_ receptors, specifically expressed on glutamatergic inputs to BLA principal neurons (Pan et al., 2009), might also reinforce the temporal precision of synaptic plasticity (Humeau et al., 2003). Alternatively, the response of axo-axonic cells might restrict principal cell firing to those most strongly excited by noxious stimuli.

The stimuli used in this study closely resemble those employed in classical fear conditioning experiments. Therefore, our results predict how BLA interneurons might be involved in fear learning. The present results were obtained from urethane-anaesthetized rats. We cannot rule out that firing patterns of BLA neurons are different in behaving animals. However, reports on responses of single units to visual or auditory cues in different brain regions and species have found strong similarities between awake and urethane anesthesia states (Niell and Stryker, 2010; Schumacher et al., 2011). Spontaneous firing frequencies appear decreased by urethane, whereas direction and magnitude of sensory-evoked responses seem unaffected. Urethane treatment induces brain states comparable to those observed in natural conditions (Clement et al., 2008). Hippocampal θ oscillations display patterns resembling those in the unanaesthetized state (Lubenov and Siapas, 2009, and our results). In addition, we found that BLA principal neurons fired similarly phase-locked to hippocampal θ as previously reported in behaving animals. In hippocampus, groups of putative interneurons recorded in behaving rats appear similarly θ-modulated to the main GABAergic cell classes recorded under urethane (Czurkó et al., 2011). Overall, it is likely that firing patterns of BLA neurons reported here recapitulate their main characteristics in drug-free conditions.

### BLA Theta Genesis

BLA-hippocampal theta synchronization increases after fear conditioning. This might facilitate the cortical transfer of emotional memories for long term storage (Paré et al., 2002; Popa et al., 2010). How may specific firings of GABAergic interneurons contribute to this? Convergent excitatory inputs onto principal cells during sensory stimuli can trigger synaptic plasticity (Humeau et al., 2003). Dendrite-targeting interneurons, such as those CB^+^ cells, could provide powerful inhibitory control of such excitatory inputs (Lovett-Barron et al., 2012). Calbindin^+^ interneurons preferentially fire before the peak of dCA1 θ. Therefore, excitatory inputs active around the θ trough are more likely to increase their synaptic weight during intense sensory stimulation.

Axo-axonic cells may ensure that synaptic potentiation is restricted to inputs concomitantly active with the salient stimulus. Assuming that some extrinsic inputs are θ-modulated, the net effect could be a stronger θ modulation of excitatory input to BLA principal neurons. This potentiation would create synchrony in large cell assemblies in synergy with the intrinsic membrane potential resonance of BLA principal neurons (Paré et al., 1995). Consistent with this, LFP θ power increases in BLA following fear conditioning (Paré and Collins, 2000; Seidenbecher et al., 2003), and BLA principal neurons become more θ modulated and synchronous after fear conditioning (Paré and Collins, 2000). These changes are made possible by the fact that in naive animals, only 20%–40% (Popa et al., 2010, and our findings) of BLA principal neurons are θ-modulated, and at dispersed phases. BLA θ oscillations increase after fear conditioning with a delay (Pape et al., 2005; Paré et al., 2002), which may be explained by the induction of structural plasticity (Ostroff et al., 2010).

The present results suggest that PV^+^ basket and axo-axonic cells play minor roles in θ increase. However, they might modify their activities with emotional learning and later support BLA θ oscillations. Futures work in behaving animals is needed to examine the activities of BLA interneurons after fear conditioning and, most critically, to address how they change during learning. Our finding of cell-type-dependent firing could be used to facilitate the classification of putative BLA interneurons recorded in behaving animals.

### Conclusion

Modulation of neuronal synchrony in the BLA is critical for the formation of emotional memories. This study provides insights into the cell type-specific contribution of GABAergic cells to BLA synchrony. Timed release of GABA on specific domains of BLA principal neuron is likely important for emotional information processing. We propose that the cooperation between precise spike-timing of various interneuron types is necessary for the encoding and persistence of emotional memories. Future studies could build on our findings to manipulate specific interneuron populations during behavior and directly test this hypothesis.

## Experimental Procedures

### In Vivo Electrophysiological Recordings

All procedures involving experimental animals were performed in accordance with the Animals (Scientific Procedures) Act, 1986 (UK) and associated regulations, under approved project and personal licenses. Seventy adult male Sprague-Dawley rats (250–350 g) were anesthetized with intraperitoneal injections of urethane (1.30 g.kg^−1^ body weight) plus supplemental doses of ketamine and xylazine, (10–15 and 1–1.5 mg.kg^−1^, respectively) as needed. The rectal temperature was maintained at 37°C with a homeothermic heating device. Craniotomies-duratomies were performed over the right hippocampus and amygdala.

Neuronal activities in the BLA and dCA1 (stratum oriens-pyramidale) were recorded with independent electrodes made of silver-chloride wires loaded in glass pipettes filled with 1.5% Neurobiotin (Vector Laboratories) in 0.5 M NaCl (12–18 MΩ resistance in vivo, tip diameter ∼1.1 μm). Glass electrode signals were referenced against a wire implanted subcutaneously in the neck. The electrocorticogram (ECoG) was recorded via a 1 mm diameter steel screw juxtaposed to the dura mater above the right prefrontal cortex (Bregma AP: 4.5 mm, ML: 2.0 mm), and was referenced against a screw implanted above the ipsilateral cerebellum.

Pinches of 15 s duration were delivered to the hindpaw controlateral to recording sites using pneumatically driven forceps that delivered a pressure of 183 g.mm^−2^. Similar mechanical stimuli have been shown to be noxious by eliciting an escape response in behaving rats, as well as by recruiting nociceptive brain circuits in urethane-anesthetized rats (Cahusac et al., 1990). Electrical stimuli (single current pulses of 5 mA intensity and 2 ms duration) were delivered at 0.5 Hz through 2 wires implanted on the ventral face of the controlateral hindpaw, for at least 100 trials. The timing of stimuli delivery was controlled by an external pulse generator (Master-8; A.M.P.I.) and synchronously recorded. Identical electrical shocks have been shown to activate spinal cord nociceptive neurons in urethane-anesthetized rats (Coizet et al., 2006).

Residual 50 Hz noise and its harmonics were reduced in all signals using Humbugs (Quest Scientific). Glass electrode signals were amplified (10×, Axoprobe 1A, Molecular Devices Inc.), bifurcated, further amplified (100×), and differentially filtered (DPA-2FS filter/amplifier; Scientifica) to extract local field potentials (LFPs, 0.3–5,000 Hz) and unit activities (300–5,000 Hz). Raw ECoG signal was band-pass filtered (0.3–1,500 Hz) and amplified (2,000×). All signals were digitized online at 16.67 kHz using a Power 1401 analog-digital converter (Cambridge Electronic Design) and stored on a PC running Spike2 software (versions 6.08 and 6.09, Cambridge Electronic Design). GABAergic cell recordings lasted 15–105 min (typically ∼45 min). The juxtacellular recording mode (rather than, for example, a quasi-intracellular mode), was assured by only including for analysis neurons that (1) had stable spontaneous firing rates/patterns and stable spike widths; (2) did not display any “injury discharge”; and (3) were recorded in the absence of spurious “baseline noise” or hyperpolarizing shifts in the electrode potential.

### Labeling of Single Neurons and Reference Sites

After recordings, neurons were selectively filled with Neurobiotin using juxtacellular labeling (Pinault, 1996). Spike shape and amplitude were monitored throughout recording and labeling to ensure that the same neuron was recorded and labeled. In order to verify the location of the reference electrode, an extracellular Neurobiotin deposit was made in the dorsal CA1 (100 nA anodal current 1 s, 50% duty cycle for 20–30 min).

### Electrophysiological Data Analysis

Only data acquired before labeling and obtained from unequivocally identified cells were analyzed. All data were analyzed off-line using Spike2 built-in functions and custom scripts (Tukker et al., 2007). Spikes were detected with an amplitude threshold in the BLA unit channel. Occasionally, additional smaller amplitude units were present in the recording. Spike2 clustering function supervised manually was used to isolate single units, and identity of labeled neurons was systematically ensured as described above. Spike sorting was always checked using autocorrelograms, which showed clear refractory periods (≥2 ms).

Hippocampal theta oscillation epochs were detected by calculating the theta (3–6 Hz) to delta (2–3 Hz) power ratio in 2 s windows of the dCA1 LFP (Csicsvari et al., 1999; Klausberger et al., 2003). Ratio >4 in at least three consecutive windows marked theta episodes. We excluded from this analysis periods of noxious stimuli and the following 20 s. Every theta episode was visually checked. Selected periods always consisted of robust theta oscillations. They exclusively occurred during persistently activated brain state (Figure S9). After theta episodes detection, the dCA1 LFP was downsampled to 1.04 kHz, digitally filtered (3–6 Hz) and the troughs were determined (Spike2). Each spike was assigned an angle relative to surrounding theta troughs (Tukker et al., 2007; Klausberger et al., 2003). The precision of our electrode placements (mediolateral and antero-posterior ranges ∼400 μm) ensured phase consistency between experiments (i.e., ∼8.5 degrees error, assuming a phase shift of 21°/mm; Lubenov and Siapas, 2009).

Mean firing frequency was calculated over 100 s continuous periods of robust theta activity. Coefficient of variation (S.D./mean, CV) of interspike intervals during these periods was used as a measure of firing regularity. CV greater than 1 indicated the cell fired in an irregular pattern.

Responses to noxious stimuli were assessed by constructing peristimulus histograms (bin size 20 ms for electrical footshocks, 200 ms for hindpaw pinches). Responses were analyzed only if the brain state corresponded to stable global activation before, during, and after the noxious stimulus. This allowed for the distinction of sensory-driven responses from effects on the brain state (e.g., change from slow wave to activation). In addition, we verified that hindpaw pinches did not induce changes in the power of the LFP oscillations recorded in dCA1 or BLA (θ and γ bands; p > 0.05, Wilcoxon signed-rank test, n = 25 cells).

### Statistical Testing

Relation to hippocampal theta oscillations: all 833–20,522 (average 6,906) spike angle values from single interneuron units were exported for testing with circular statistics (Oriana v. 2.0, Kovac Computing Services). Modulation in phase with dCA1 theta oscillations was tested for significance using Rayleigh's uniformity test (significance p < 0.005). If p < 0.005, the sum vector of all spikes was computed and normalized by the number of spikes. Its orientation determined the mean angle of spike firing, with respect to the trough (0°) of dCA1 theta oscillation (180° represents the theta peak). The length r of the normalized vector determined modulation depth. Phase modulation homogeneity within neuron groups (only modulated cells included) was tested with Moore's non parametric test (Zar, 1999). The null hypothesis was the absence of directionality in the group. If p < 0.05, cells of the group fired at consistent phases and Batschelet's method was used to calculate the population mean angle (Zar, 1999). This ensured the statistical reliability of our conclusions on population modulation. Furthermore, we established that the depth of modulation of BLA interneurons activity was not correlated with either the power or the mean frequency of dCA1 theta oscillations (Pearson correlation, R = 0.03, p = 0.896; R = 0.216, p = 0.335; respectively, n = 22).

Significance of responses to noxious stimuli was tested using thresholds. Footshocks: significance was accepted if at least 3 consecutive bins differed from the preonset 300 ms mean by 2 SD or any bin by 4 SD. Pinches: for 1–2 trials, significance was accepted if at least 3 consecutive bins differed from the preonset 10 s mean by 1 SD or any 1 bin by 4 SD. For 3 trials and more, significance was accepted if at least 3 consecutive bins differed from the preonset mean by 1.5 SD or any 1 bin by 4 SD. Latency was defined as the starting time of the first bin meeting these criteria. The peak time was the starting time of the largest change in the first significant series.

Differences in postsynaptic dendrite diameter between cell subgroups were evaluated using the Kruskal-Wallis test followed by Dunn's post hoc analysis.

Data are expressed as mean ± SEM, unless otherwise stated.

### Tissue Processing and Anatomical Analysis

Details on brains fixation, immunofluorescence, electron microscopy, and camera lucida reconstructions are given in the Supplemental Information.

## Figures and Tables

**Table 1:** Spontaneous Firing of the GABAergic Interneurons Recorded In Vivo

Recorded neurons		Spontaneous Firing during dCA1 Theta					
Type	Cell Code	Rate (Hz)	CV	Mean Angle	Angular Deviation	p (Rayleigh)	Modulation Depth
Axo-axonic	tjx20f	6.5	1.41	n.s.	n.s.	1.7 × 10^−1^	n.s.
	tjx27b	15.9	0.82	n.s.	n.s.	3.5 × 10^−1^	n.s.
	tjx56b	7.9	1.00	187.0	137.5	2.2 × 10^−6^	0.06
	tjx63a	15.6	1.02	251.5	156.0	3.7 × 10^−3^	0.02
	tjx66a	13.0	1.28	213.0	127.0	4.3 × 10^−41^	0.09
	tjx74a	15.4	0.97	283.7	146.5	1.1 × 10^−7^	0.04
PV+ basket	tjx38a	23.1	0.66	n.s.	n.s.	5.5 × 10^−3^	n.s.
	tjx41c	23.7	0.69	357.5	148.1	3.9 × 10^−5^	0.04
	tjx42b	16.7	0.67	173.9	141.8	2.2 × 10^−10^	0.05
	tjx43a	6.4	0.93	n.s.	n.s.	1.4 × 10^−1^	n.s.
	tjx48a	27.2	0.61	14.7	150.2	1.9 × 10^−7^	0.03
	tjx49a	3.9	1.18	83.2	133.7	4.0 × 10^−8^	0.07
	tjx53a	6.8	1.97	150.1	163.9	3.2 × 10^−3^	0.02
	tjx55b	9.4	1.03	13.2	113.3	3.7 × 10^−44^	0.14
	tjx61a	8.6	1.25	125.1	141.7	5.1 × 10^−8^	0.05
	tjx69a	12.2	0.94	136.5	156.7	1.4 × 10^−4^	0.02
	tjx72b	4.3	1.27	140.8	146.2	1.5 × 10^−5^	0.04
	tjx72d	1.8	1.28	97.8	130.2	1.3 × 10^−6^	0.08
	tjx78a	6.2	0.97	339.9	108.4	4.1 × 10^−38^	0.17
	tjx86b	10.4	0.96	n.s.	n.s.	1.5 × 10^−2^	n.s.
	tjx87b	4.9	1.00	183.5	145.5	5.7 × 10^−9^	0.04
CB+ dendrite-targeting	tjx21i	4.3	0.96	145.0	127.5	5.1 × 10^−13^	0.08
	tjx22c	3.1	1.00	145.5	112.9	1.8 × 10^−31^	0.14
	tjx59b	3.0	1.03	144.3	110.6	6.8 × 10^−20^	0.16
AStria-projecting	tjx45a	3.4	1.54	n.s.	n.s.	7.9 × 10^−1^	n.s.
	tjx52a	6.0	1.15	155.7	111.5	3.6 × 10^−42^	0.15
	tjx68a	4.1	0.98	158.2	122.0	7.3 × 10^−13^	0.10
	tjx83c	4.6	1.60	284.9	122.9	6.3 × 10^−10^	0.10

CV: coefficient of variation of interspike intervals (variance/mean); n.s.: not statistically significant.

**Table 2:** Noxious Stimulus-Driven Firing of the GABAergic Interneurons

Recorded Neurons		Hindpaw Pinch Responses				Electrical Footshock Responses			
Type	Cell Code	Response	Latency (ms)	Peak (ms)	%	Response	Latency (ms)	Peak (ms)	%
Axo-axonic	tjx20f	E	200	600	378	n.t.	n.t.	n.t.	n.t.
	tjx27b	E	200	400	305	E	40	260	299
	tjx56b	E	200	400	606	E	100	200	194
	tjx63a	E-I	200-1120	600-1120	133-100	n.t.	n.t.	n.t.	n.t.
	tjx66a	E	400	600	595	E	40	420	239
	tjx74a	E	400	400	246	E-I-E	20-40-140	20-60-220	172-81-213
PV+ basket	tjx38a	E	1200	2800	125	n.t.	n.t.	n.t.	n.t.
	tjx41c	I	9600	9800	55	I	100	100	38
	tjx42b	E-I	200-1040	200-1060	130-72	I	100	240	45
	tjx43a	n.t.	n.t	n.t.	n.t.	n.t.	n.t.	n.t.	n.t.
	tjx48a	I	5000	5000	27	E	20	20	71
	tjx49a	E	600	2400	218	n.s.	n.s.	n.s.	n.s.
	tjx53a	n.s.	n.s.	n.s.	n.s.	E	20	100	115
	tjx55b	E	200	8800	526	I	180	220	68
	tjx61a	E	600	1600	153	n.s.	n.s.	n.s.	n.s.
	tjx69a	E-I	200-1600	200-2800	160-100	n.s.	n.s.	n.s.	n.s.
	tjx72b	I	5000	5000	100	I	340	400	100
	tjx72d	n.t.	n.t.	n.t.	n.t.	n.t.	n.t.	n.t.	n.t.
	tjx78a	E	600	600	314	E	40	180	170
	tjx86b	E	2200	2200	62	n.t.	n.t.	n.t.	n.t.
	tjx87b	n.t.	n.t.	n.t.	n.t.	I	100	200	72
CB+ dendrite-targeting	tjx21i	I	4200	4400	100	n.t.	n.t.	n.t.	n.t.
	tjx22c	n.t.	n.t.	n.t.	n.t.	n.t.	n.t.	n.t.	n.t.
	tjx59b	n.s.	n.s.	n.s.	n.s.	n.t.	n.t.	n.t.	n.t.
AStria-projecting	tjx45a	I	1000	1000	100	I	60	740	100
	tjx52a	I	1600	1800	100	n.t.	n.t.	n.t.	n.t.
	tjx68a	n.s.	n.s.	n.s.	n.s.	I	20	20	75
	tjx83c	I	3800	3800	100	I	20	380	81

%: maximal percentage of sensory-evoked firing changes; E: excitation; I: inhibition; n.s.: not statistically significant; n.t.: not tested.

## Supplemental Information

Document S1. Figures S1–S9, Tables S1–S8, and Supplemental Experimental Procedures
