# Supplementary material for: Cell-Type-Specific Recruitment of Amygdala Interneurons to Hippocampal Theta Rhythm and Noxious Stimuli In Vivo
Source: Neuron. 2012 Jun 21;74-20(6):1059–74. doi: 10.1016/j.neuron.2012.04.022 (PMC3391683; doi:10.1016/j.neuron.2012.04.022)
Supplement: Document S1. Figures S1–S9, Tables S1–S8, and Supplemental Experimental Procedures [file mmc1.pdf]

Supplemental Information

Cell-Type-Specific Recruitment of Amygdala Interneurons to Hippocampal Theta Rhythm and Noxious Stimuli In Vivo

Thomas C.M. Bienvenu, Daniela Busti, Peter J. Magill, Francesco Ferraguti, and Marco Capogna

INVENTORY OF SUPPLEMENTAL INFORMATION

Contains 9 Supplemental Figures, 7 Supplemental Tables, Supplemental Experimental Procedures, and Supplemental references.

- **Figure S1, linked to Figures 1, 2, 3, 4 and 6**: Somata locations of the recorded neurons in the basolateral amygdala. This figure shows that neurons were recorded throughout the BLA, and that subnuclei specificities cannot account for functional heterogeneities among cells of a type.

- **Figure S2, linked to Figures 1-5**: Firing relationships of individual BLA interneurons with hippocampal theta oscillations.

- **Figure S3, linked to Figures 1-4:** Firing relationships of BLA interneuron types with hippocampal gamma oscillations**.** This figure shows that none of the four interneuron types displayed firing modulation by dCA1 gamma oscillations (30-80 Hz).

- **Figure S4, linked to Figures 1-5**: Firing responses of individual BLA interneurons to hindpaw pinches.

- **Figure S5, linked to Figures 1-5**: Firing responses of individual BLA interneurons to electrical footshocks.

Figures S2, S4 and S5 show the electrophysiological results obtained for each interneuron, and illustrate how the main conclusions were reached.

- **Figure S6, linked to Figures 2-4**: Postsynaptic target analysis. This figure illustrates extensively the method used to identify postsynaptic targets of BLA interneurons at electron microscopic level. In panel (E), the diameters of dendrites targeted by various cell types are compared.

- **Figure S7, (A) linked to Figure 2, (B) linked to Figure 4**: Rostro-caudal extent of BLA interneurons. Represents for the first time entire views of BLA interneurons, an information necessary to unambiguously define cell types.

- **Figure S8, linked to Figure 6:** Extracellular spike waveform analysis. This figure shows that the four types of BLA interneurons could not be separated on the basis of their spike shapes. In contrast, GABAergic interneurons fired shorter action potentials than principal glutamatergic cells, as confirmed with unsupervised cluster analysis.

- **Figure S9, linked to Figure 7**: Spectral characteristics of local field potentials recorded in dorsal and ventral hippocampus and amygdala. This figure shows that LFP theta oscillations could be recorded in dCA1 and ventral hippocampus, but not in amygdala. Theta oscillations were robust and continuous only in dCA1.

- **Table S1, linked to Figures 1-4**: Neuronal domains innervated by interneurons of the BLA. This table contains detailed results of the electron microscopic analysis.

- **Table S2, linked to Figures 1-4**: Postynaptic targets and neurochemical content of *in vivo*-recorded GABAergic neurons. This table gives detailed results of light microscopic analysis.

- **Table S3, linked to Figures 1-4 and Figure S3**: Analysis of dCA1 gamma modulation of BLA interneuron's spiking. This table contains the results obtained for each interneuron.

- **Table S4, linked to Figure 6**: Principal cells: electrophysiological and immuno-cytochemical analysis. This table contains results of immunohistochemical and electrophysiological analysis for each principal neuron.

- **Table S5, linked to Figure 6 and Figure S8**: Spike waveform parameters of all GABAergic and principal neurons. This table summarizes the values measured for every parameter in each of the 51 cells reported in this paper.

- **Table S6, linked to Figure 7**: Theta phase modulation of BLA neuron firing assessed with dorsal and ventral hippocampal references. This table recapitulates how the data plotted in Figure 7D were obtained. Moreover, it shows that LFP theta phase in ventral hippocampus varies between experiments by as much as 68.3˚ relative to dCA1 theta.

- **Table S7**: Primary antibodies used. This table explains how antibodies were obtained, diluted, and their specificity verified.

Supplemental Experimental Procedures describe recordings and analyses of ventral hippocampal theta and BLA principal neurons, anatomical methods, as well as the procedures followed to study CA1 gamma oscillations and spike waveforms.

Somata locations of the recorded neurons in the basolateral amygdala.

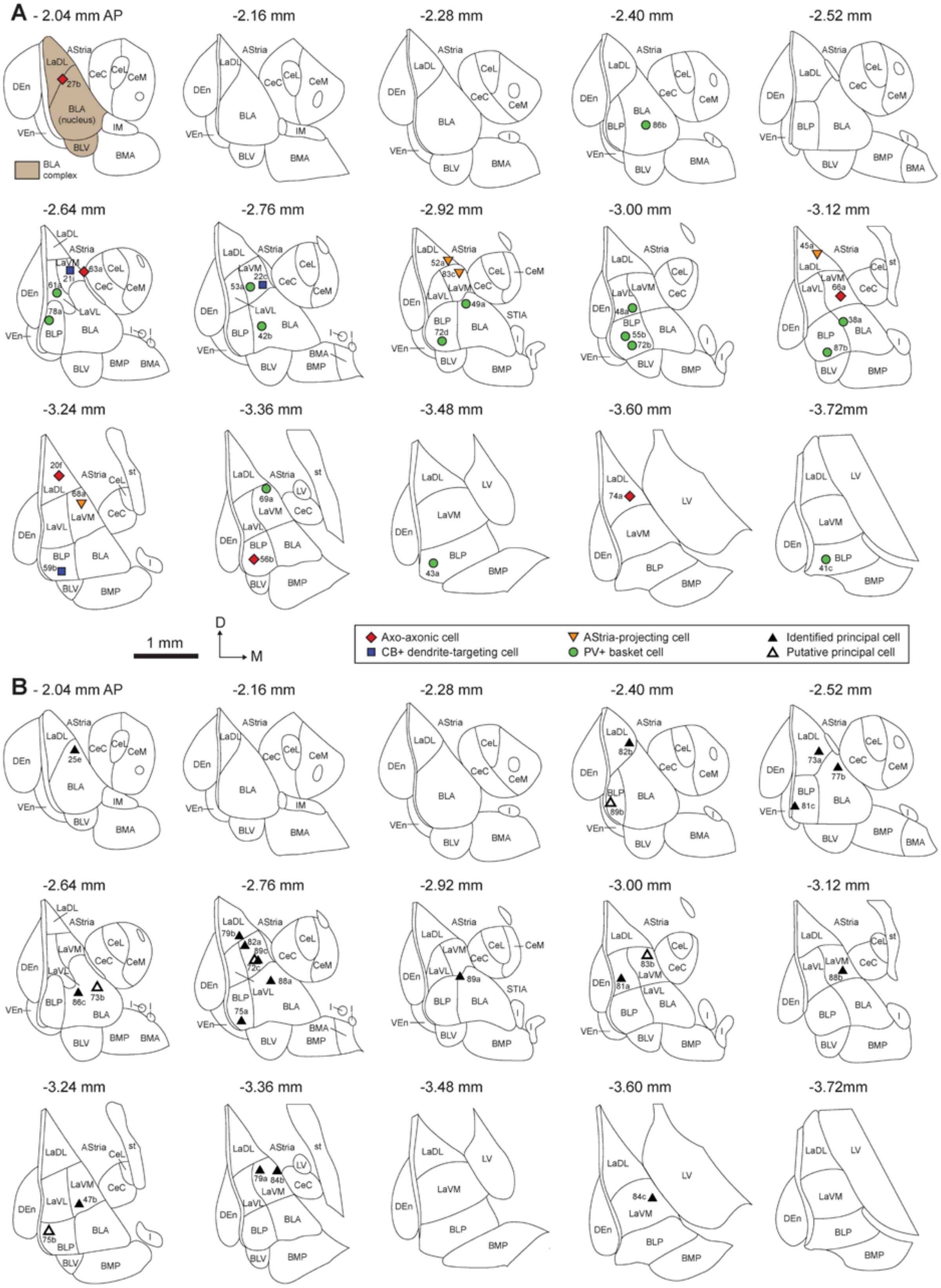

Figure S1. Somata locations of the recorded neurons in the basolateral amygdala (related to Figures 1-4 and 6).

Each symbol represents the position of a recorded neuron's soma in schematic coronal sections of the rat amygdala. The prefix "tjx" was removed from the experimental codes of neurons for clarity. Nomenclature and anatomical boundaries follow those of the *Rat brain in stereotaxic coordinates* (Paxinos and Watson, 2007). Distances above each panel represent the antero-posterior position (AP) of the corresponding coronal plane relative to the bregma junction (according to Paxinos and Watson, 2007). **(A)** Positions of the anatomically-identified GABAergic cells' somata. Except the basal ventral nucleus (BLV), our recordings covered all subdivisions of the amygdaloid basolateral complex. **(B)** Locations of the anatomically-identified and putative principal glutamatergic neurons recorded. Positions of putative principal neurons were extrapolated back from the documented distance to a labeled neuron in the same electrode track. D: dorsal, M: medial. Adapted with permission from Paxinos and Watson (2007).

Firing relationships of individual BLA interneurons with hippocampal theta oscillations.

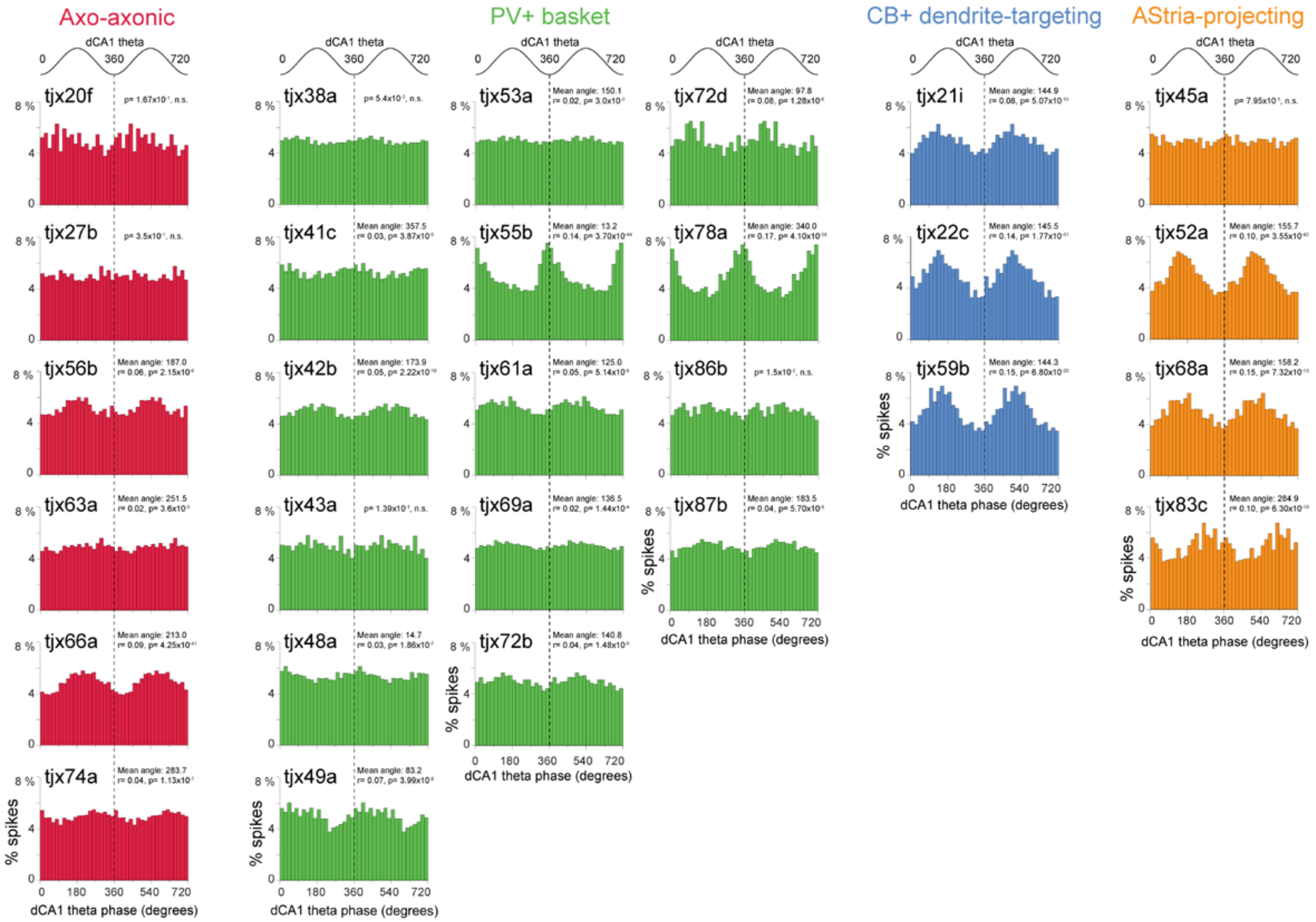

Figure S2. Firing relationships of individual BLA interneurons with hippocampal theta oscillations (related to Figures 1-5).

Phase histograms showing the spike distribution of each interneuron relative to hippocampal theta cycles, constructed from the entire dCA1 theta oscillations periods. Some neurons displayed a strong preference for a specific phase of the theta cycle (e.g., the CB+ dendrite-targeting cell tjx22c). The firing of some neurons showed only a small coupling to the theta rhythm (e.g., the PV+ basket cell tjx48a). The firing of other neurons was not significantly modulated (e.g. the axo-axonic cell tjx27b).

Homogeneity of phase locking was only observed for CB+ dendrite-targeting cells, a finding confirmed by statistical analysis (Moore test, see Results). In contrast, heterogeneity was observed for the other interneuron types, e.g. in PV+ basket cells. Mean angle, p values (Rayleigh test) and modulation depths are indicated for each cell. Two theta cycles are represented for clarity. 0˚, 360˚ and 720˚: theta troughs. Bin size of the histograms: 18°.

Firing relationships of BLA interneuron types with hippocampal gamma oscillations.

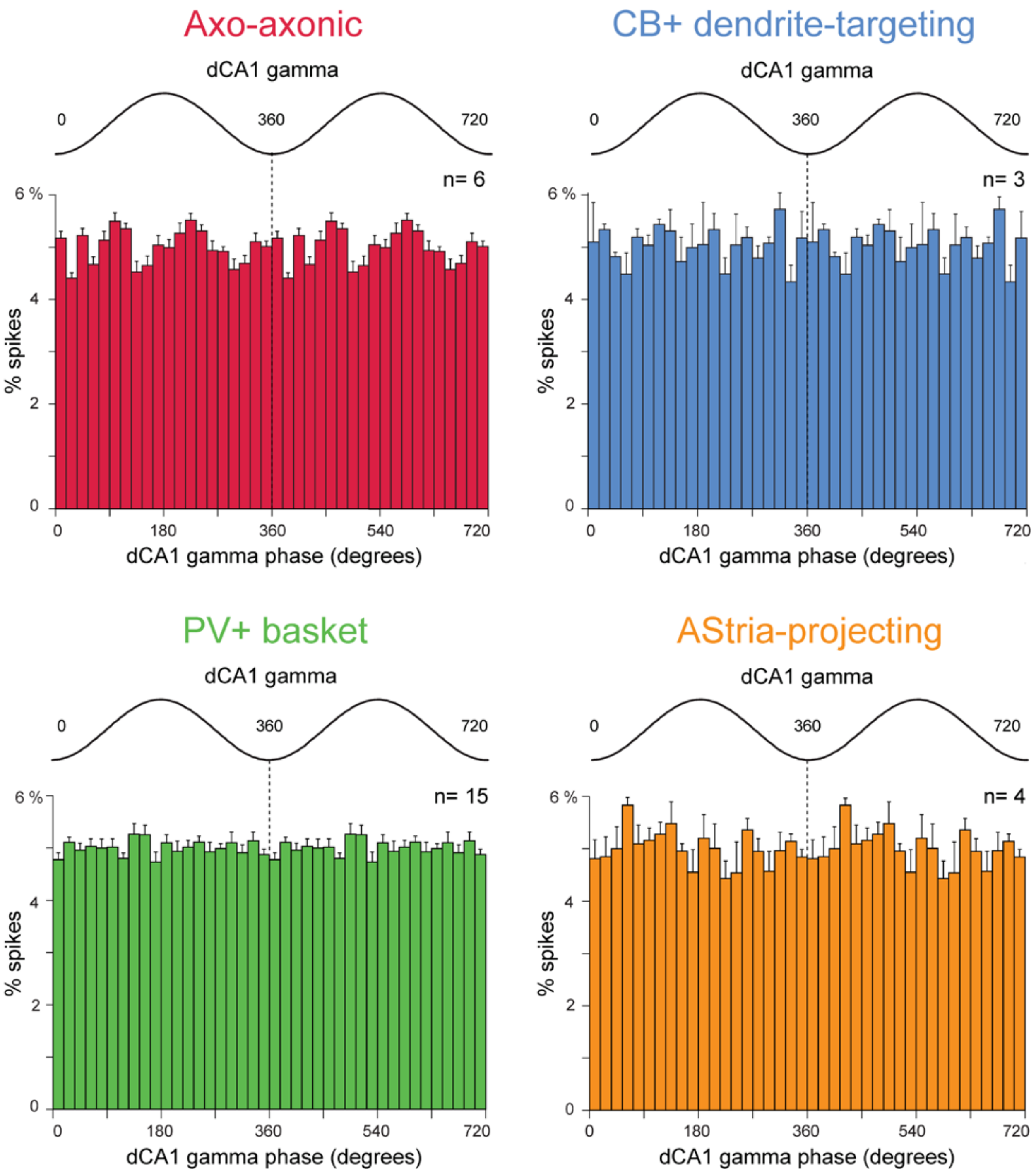

Figure S3. Firing relationships of BLA interneuron types with hippocampal gamma oscillations (related to Figures 1-4).

The firing of BLA interneurons is not modulated in phase with dCA1 gamma oscillations. This figure shows results obtained with the analysis of gamma oscillations (30-80 Hz) nested in dCA1 theta oscillations (3-6 Hz). Phase histograms (18° bins) represent mean spike timing of BLA interneuron types relative to gamma oscillation troughs (detected in dCA1 stratum oriens / pyramidale). Spike timing probability (%) in phase histograms was computed by dividing the number of action potentials in each bin by the total number of action potentials. No significant modulation was observed for the 28 interneurons (Rayleigh test). Similar results were obtained when analyses were performed on all gamma oscillation periods (regardless of their relationship to the theta rhythm). None of the 28 interneurons recorded in this study showed statistically significant modulation in phase with dCA1 gamma oscillations ($p > 0.005$, Rayleigh test). This held true for the analysis of theta-nested gamma oscillations, and for entire gamma oscillation periods (Table S3). This finding is consistent with gamma oscillations being generated locally. Error bars: SEM.

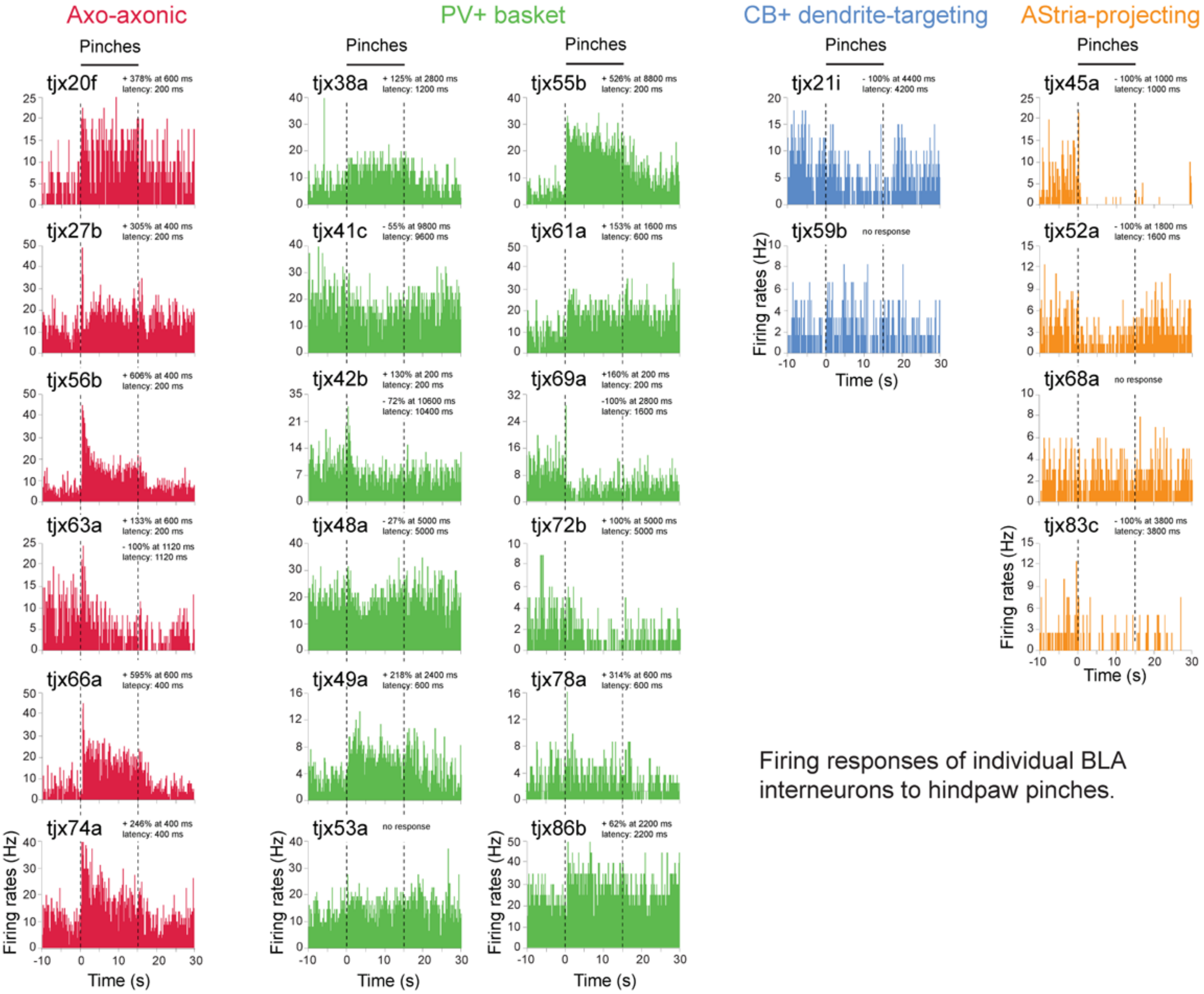

Firing responses of individual BLA interneurons to hindpaw pinches.

Figure S4. Firing responses of individual BLA interneurons to hindpaw pinches (related to Figures 1-5).

Peristimulus histograms showing spiking frequencies before, during and after hindpaw pinches for each interneuron recorded. Axo-axonic neurons and AStria-projecting cells responded relatively homogeneously to hindpaw pinches. In contrast, PV+ basket cells displayed heterogeneous responses, as recapitulated in their group average (see Figure 5). Latency of responses, and percentage and time of occurrence of maximal firing changes after pinches are indicated for each graph. Bin size of the histograms: 200 ms.

Firing responses of individual BLA interneurons to electrical footshocks.

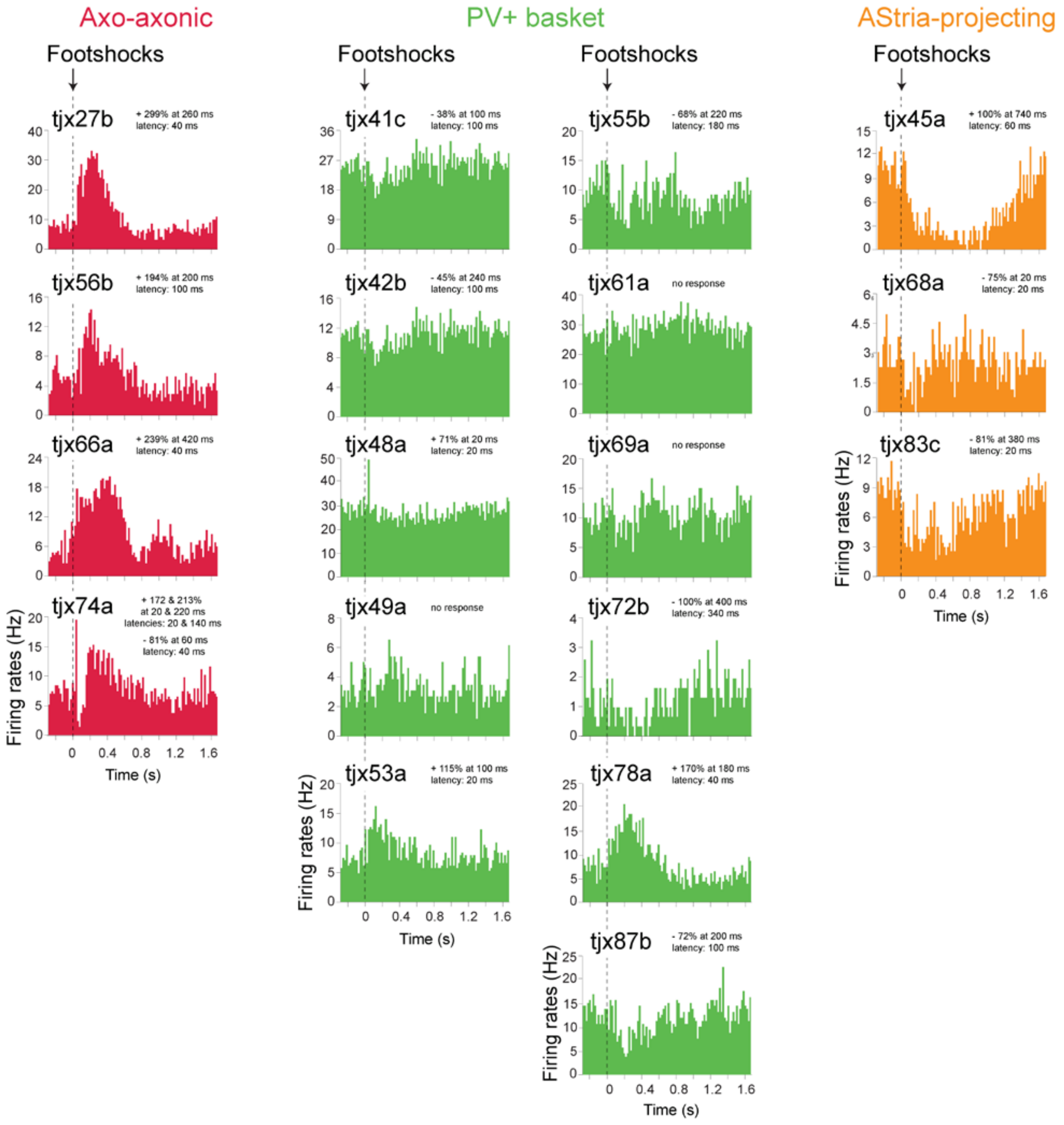

Figure S5. Firing responses of individual BLA interneurons to electrical footshocks (related to Figures 1-5).

Peristimulus histograms showing spiking frequencies before, during and after electrical footshocks for each interneuron recorded. Response patterns were similar to those observed with hindpaw pinches. All axo-axonic cells increased their firing frequency upon electrical footshocks, AStria-projecting cells were inhibited, whereas PV+ basket cells responded moderately and heterogeneously. Percentage, latency and time of occurrence of maximal firing changes after footshocks are indicated for each graph. Bin size of the histograms: 20 ms.

Postsynaptic target analysis.

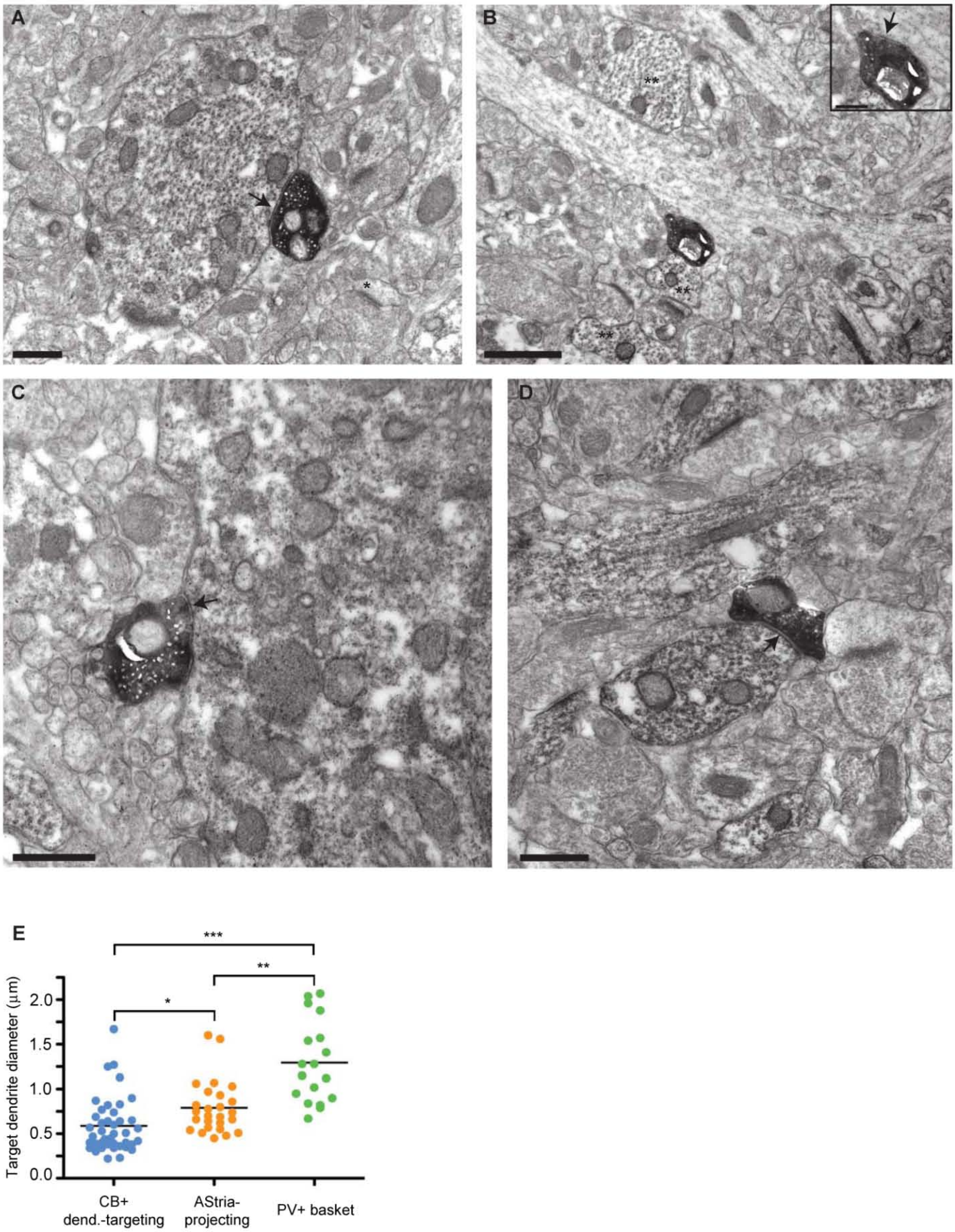

Figure S6. Postsynaptic target analysis (related to Figures 2-4).

Axonal boutons of Neurobiotin-filled neurons were visualized by HRP reaction using nickel-intensified DAB as a chromogen. This reaction resulted in a highly electron-opaque cytoplasmic precipitate sparing synaptic vesicles and mitochondria. Arrows indicate synaptic junctions. To determine if postsynaptic profiles were processes of glutamatergic principal neurons, we used anti-CaMKIIα immunoperoxidase. This method appears specific and sensitive for principal neurons (see Supplemental Experimental Procedures). However, many more small dendrites and dendritic spines than expected were unlabeled (potentially false negatives; see panels A and B; *: unlabeled spine making an asymmetrical synapse with a nearby axonal bouton). Thus, under our experimental conditions, the lack of CaMKIIα immunoperoxidase labeling did not allow to exclude that the postsynaptic target was a principal cell. As a possible explanation, we observed that some anatomically-identified glutamatergic neurons expressed low levels of CaMKIIα (Figure 6C), that could not be detected in their dendrites using immunofluorescence.

(A) Symmetric (Gray's Type-II) synaptic junction formed by a PV+ basket cell (tjx49a) bouton with a large dendrite of a CaMKIIα+ neuron in BLA, identified by a fine electron-opaque HRP reaction product (using non-intensified DAB as chromogen).

(B) Type-II synaptic junction made by another bouton of tjx49a with a dendrite devoid of visible DAB product. Such postsynaptic target neuron remained unidentified. It could correspond to another GABAergic cell. Note the presence of clear DAB staining in nearby dendrites (**). The inset shows the synapse at higher magnification.

(C) Type-II synaptic junction formed by a third axonal bouton of tjx49 with the soma of an identified CaMKIIα+ principal neuron.

(D) Type-II synapse made by a bouton of an AStria-projecting neuron (tjx68a) with a dendrite of a CaMKIIα+ in the AStria. This target cell was a putative medium-sized spiny neuron, as we observed robust co-expression of CaMIIα and DARPP-32 in the AStria (see Figure 4E).

(E) Diameters of dendrites targeted by different interneuron types in the BLA. Circles represent individual measurements; horizontal bars represent mean values. Group differences were analyzed by the Kruskal-Wallis test followed by Dunn's post hoc analysis. *, $p < 0.05$, ** $p<0.01$, *** $p<0.005$.

Scale bars: (A) 1 μm; (B) 1 μm, inset: 250 nm; (C,D) 500 nm.

Rostro-caudal extent of single BLA interneurons.

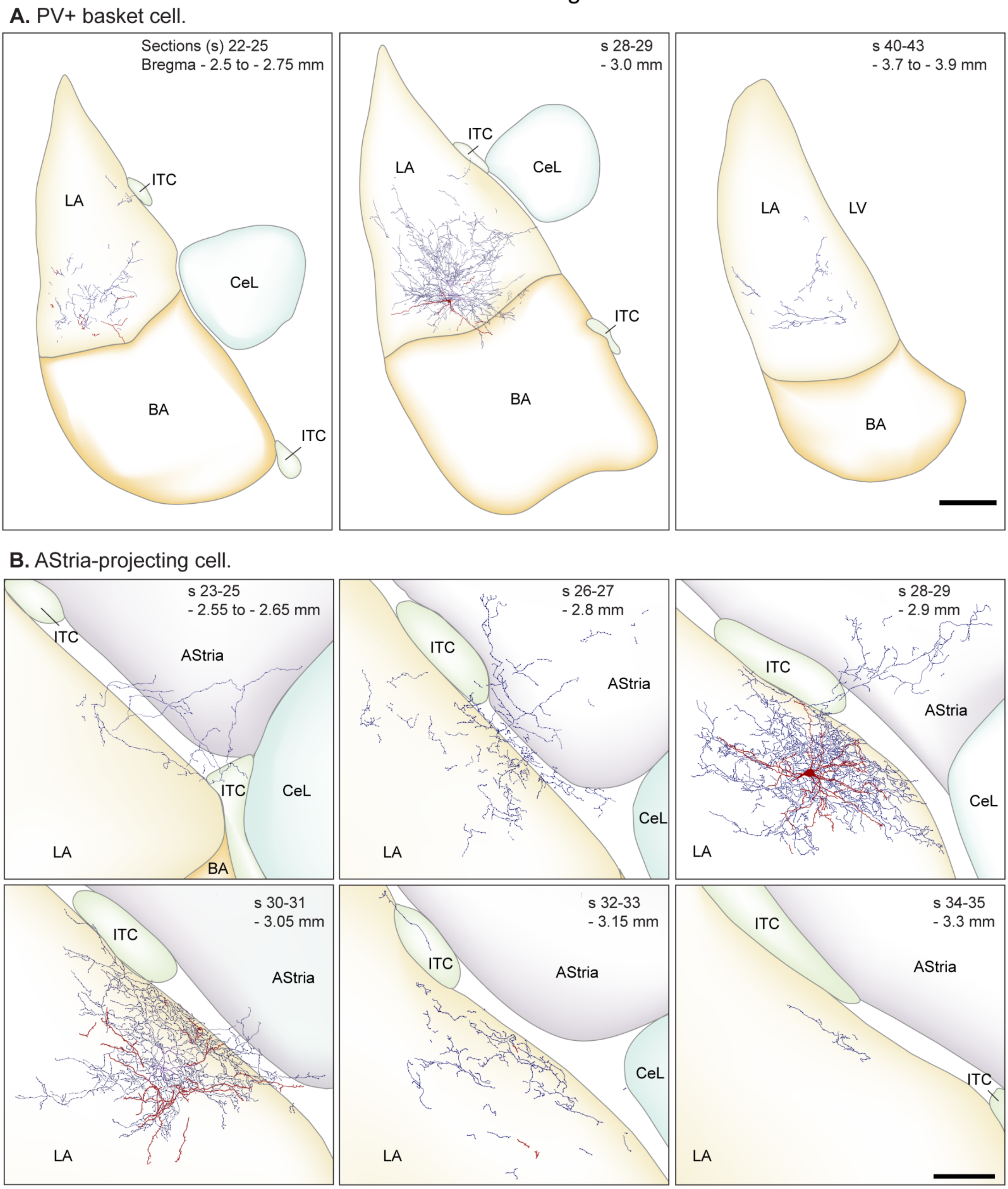

Figure S7. Rostro-caudal extent of single BLA interneurons (related to Figures 2 and 4).

Each panel shows the camera lucida reconstruction of dendrites (red) and axons (blue) from the corresponding subset of 60 μm-thick sections, at the indicated antero-posterior level (relative to bregma; following Paxinos and Watson, 2007).

(A) PV+ basket cell tjx48a (same cell as in Figure 2). The left panel shows the most rostral processes. The central panel depicts the section containing the soma and the section immediately caudal to it. The right panel illustrates the four sections before the last containing filled axon. The axon and dendrites of this cell covered a large portion of the BLA, in particular the lateral nucleus (axonal extent 1.4 mm anteroposterior). This example neuron was not unusual but in the upper size range. Such PV+ basket cells were the largest interneurons found in this study in terms of both dendritic and axonal spans. Dendritic and axonal trees of PV+ basket cells were stereotyped morphologically but varied proportionally in size. Large and small PV+ basket cell subtypes might exist, but we rather observed a size continuum (as suggested by McDonald and Betette, 2001).

(B) Reconstruction of the full dendritic and axonal fields of an AStria-projecting neuron (tjx52a; same cell as in Figure 4) from 13 sections (s23-s35; axonal extent: 780 μm rostrocaudal). In contrast to PV+ basket cells, dendrites of AStria-projecting cells never extended far from the soma. Likewise, their local axons were mostly confined to the dendritic field and were extraordinarily dense. Note that the projection to AStria was not merely the result of one axonal branch overlapping with BLA boundaries. On the contrary, it represented a significant part of the axonal arborisation and could be observed at several antero-posterior levels. An even larger axonal projection to AStria was observed in one neuron (tjx68a).

AStria: amygdala-striatum transition area, BA: basal amygdala, CeL: central lateral amygdaloid nucleus, ITC: intercalated cells cluster, LA: lateral amygdala, LV: lateral ventricle. Scale bars: (A) 250 μm, (B) 100 μm.

Extracellular spike waveform analysis.

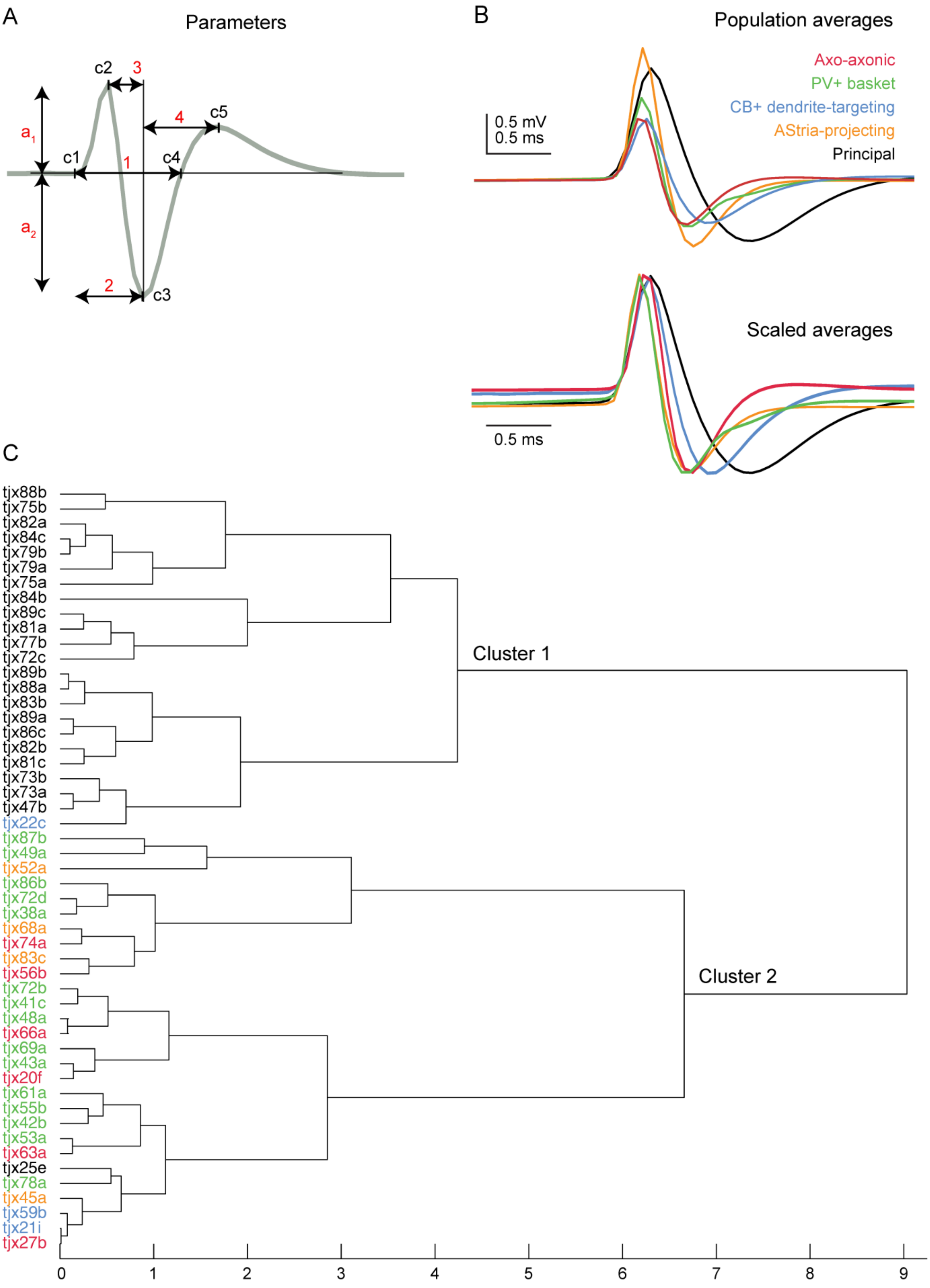

Figure S8. Extracellular spike waveform analysis (related to Figure 6).

This work identified for the first time BLA glutamatergic cells and interneurons recorded *in vivo*. Taking advantage of this, we investigated whether cell classes could be separated on the basis of their spike waveforms.

(A) Schematic of the parameters measured for each cell. 1: total width (c1-c4), 2: baseline to trough (c1-c3), 3: first peak to trough (c2-c3), 4: trough to second peak duration (c3-c5); a_1/a_2: first peak / trough amplitude ratio. Cursors (c1-5) were placed as indicated in Supplemental Procedures. Measures made for each neuron are recapitulated in Table S5.

(B) Average spike waveform of each neuron class. Top: raw data, bottom: amplitude-scaled traces. Principal neurons fired longer-lasting spikes than interneurons as a group. Except trough to second peak duration, all spike width parameters were smaller for interneurons ($p < 0.0001$, Mann-Whitney U-test). The 1st peak/trough amplitude ratio did not differ between interneurons and principal cells ($p > 0.1$, Mann-Whitney U-test). None of the five parameters analyzed showed significant differences across interneuron types ($p > 0.05$, Kruskal Wallis test).

(C) Representative cluster analysis of spike waveforms. Cluster analyses using the various spike waveform parameters isolated two clusters, formed by interneurons and principal cells (here: cluster 1 and cluster 2, respectively; only one principal cell in the interneuron cluster and vice versa). They also confirmed the lack of statistical separation across interneuron types. X-axis shows the squared Euclidian distance between group centroids. Colour code is the same as elsewhere in the paper.

Our data suggest that spike shapes can be used to distinguish BLA interneurons and principal cells recorded extracellularly *in vivo*. This important issue has remained a matter of debate so far, in the absence of unambiguous identification of the recorded neurons. The present results are consistent with an earlier work proposing a spike duration cut-off of 0.5 ms (measured from peak to trough) for putative BLA interneurons ((Likhtik et al., 2006), Table S5). However, we cannot exclude that BLA interneuron types we did not record might fire longer spikes.

Spectral characteristics of local field potentials recorded in dorsal and ventral hippocampus and amygdala.

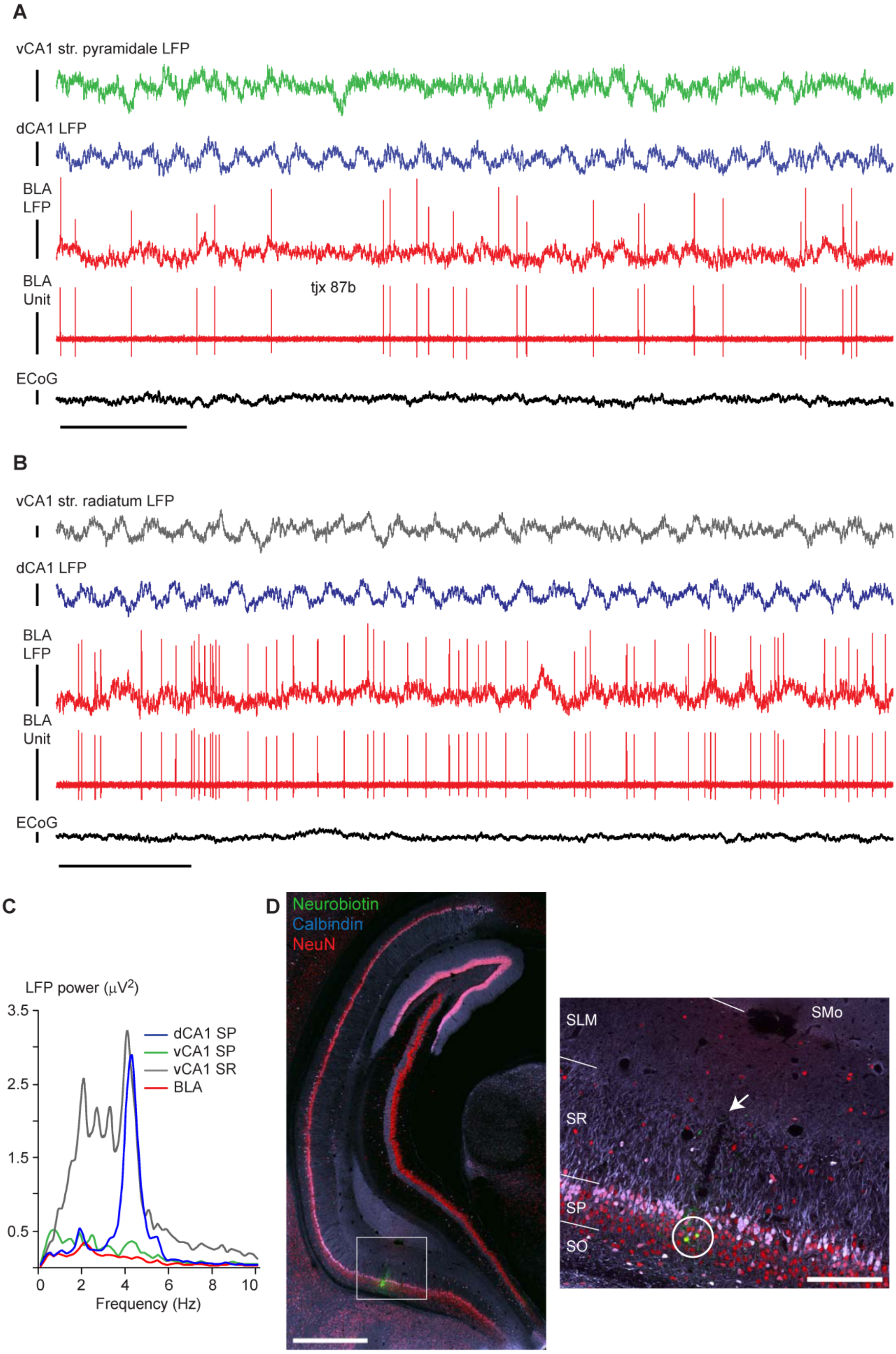

Figure S9. Spectral characteristics of local field potentials recorded in dorsal and ventral hippocampus and amygdala (related to Figure 7).

This figure shows typical data obtained with triple BLA, dCA1 and vCA1 recordings. In this experiment, LFP references were recorded in dorsal and ventral hippocampus, and a PV+ basket cell (tjx87b) was recorded in the BLA, during cortical activation (characterized by low amplitude, high frequency oscillations in the ECoG signal (Mallet et al., 2008)). No prominent theta oscillations were observed in str. pyramidale of vCA1 or in the BLA LFPs. In contrast, intermittent, but large amplitude theta oscillations could be recorded in vCA1 str. radiatum.

(A,B) Original data. LFPs: 0.3-300 Hz in this experiment. In A, the vCA1 reference electrode was located in str. pyramidale. **(B)** Keeping the single BLA unit recording stable, the vCA1 electrode was moved to str. radiatum (retracted by 355 μm).

(C) Corresponding power spectra of LFP signals. The dCA1 LFP shows a clear peak at ~4 Hz, indicating robust theta oscillations. LFPs of vCA1 str. pyramidale and BLA have no power peak in the theta range (3-6 Hz). LFP of vCA1 str. radiatum displays peaks at ~4 Hz, as well as lower frequencies (consistent with intermittent theta oscillations). SP: str. pyramidale: SR, str. radiatum.

(D) Structured illumination images showing the anatomically-confirmed recording sites in vCA1 from the same experiment. The white circle highlights the Neurobiotin (green) deposit made at the str. pyramidale recording site. NeuN immunoreactivity (red) was used as a pan-neuronal nuclear and cytoplasmic marker to delineate str. pyramidale. Calbindin immunoreactivity (light blue) highlights hippocampal layers. Colocalization with NeuN is indicated by white and pink. Right: higher magnification of the area delineated in the left panel. Arrow: recording site in str. radiatum, close to the str. lacunosum moleculare border. SO: str. oriens; SP: str. pyramidale: SR, str. radiatum; SLM: str. lacunosum moleculare; SMo: str. moleculare, dentate gyrus. Scale bars: (A,B) time: 1 s, LFPs: 0.5 mV, ECoG: 0.25 mV, unit tjx87: 1.5 mV; (D) left: 1 mm, right: 200 μm.

**Cell type**	**Axo-axonic**		**PV+ basket**		**CB+ dendrite-targeting**		**AStria-projecting**	
**Cell code** / **Postsynaptic target** (*CaMKIIα+*)	tjx20f	tjx66a	tjx43a	tjx49a	tjx22c	tjx59b	tjx52a	tjx68a
Soma	-	1	8 (*7*)	14 (*12*)	-	-	11 (*1*)	3 (*2*)
Dendrite	-	-	12 (*7*)	6 (*3*)	21 (*5*)	20 (*5*)	9 (*4*)	17 (*7*)
Axon initial segment	21	20*	-	-	-	-	-	-
Unidentified	-	1**	-	-	-	-	-	-
Total	21	22	20	20	21	20	20	20

Table S1. Neuronal domains innervated by interneurons of the BLA.

Postsynaptic target identity was determined using standard criteria (Peters et al., 1991). Synapses were analyzed in serial (3-10) ultrathin sections. The table indicates the number of synapses found in the corresponding categories.

* One synapse was made with a dendritic axon hillock (segment from which emerged the axon initial segment, as defined in Peters et al., 1991).

** Since four investigators could not conclude whether the postsynaptic profile was part of an AIS or a dendrite, it was considered unidentified.

Recorded neurons		Postsynaptic targets				Molecular markers									
Cell type	Cell code	Ankyrin G+	CamKIIα+ s and d	DARPP-32+	EM	Calbindin	CB1-R	Calretinin	$GABA_A$-Rα1	GAD	Kv 3.2	Nk1-R	PV	SOM	VGAT
Axo-axonic	tjx20f	AIS	n.t.	-	AIS	0 (d)	n.t.	n.t.	0 (s,d)	n.t.	n.t.	n.t.	1 (s,d)**	0 (s)	n.t.
	tjx27b	AIS	n.t.	-	n.t.	0 (d)	n.t.	0 (d)	0 (s,d)	n.t.	n.t.	n.t.	1 (d,ax)	n.t.	n.t.
	tjx56b	AIS	0	-	n.t.	0 (s)	n.t.	n.t.	1 (d)	n.t.	n.t.	n.t.	1 (d,ax)	n.t.	n.t.
	tjx63a	AIS	n.t.	-	n.t.	0 (d)	n.t.	n.t.	0 (d)	n.t.	n.t.	n.t.	1 (s,d,ax**)	n.t.	n.t.
	tjx66a	AIS	n.t.	-	AIS	0 (d)	n.t.	n.t.	1 (d)	n.t.	1 (s)	n.t.	1 (s,d,ax)	n.t.	n.t.
	tjx74a	AIS	n.t.	-	n.t.	0 (d)	n.t.	n.t.	0 (d)	n.t.	n.t.	n.t.	1 (d,ax)	n.t.	n.t.
PV+ basket	tjx38a	n.t.	n.t.	-	n.t.	1 (d)	n.t.	n.t.	1 (d)	n.t.	n.t.	n.t.	1 (d)	n.t.	n.t.
	tjx41c	n.t.	s, d	-	n.t.	1 (d)**	n.t.	n.t.	1 (d)	n.t.	n.t.	n.t.	1 (d,ax)	n.t.	n.t.
	tjx42b	n.t.	D, s	-	n.t.	1 (d,ax)	n.t.	n.t.	1 (d)	n.t.	n.t.	n.t.	1 (d,ax)	n.t.	n.t.
	tjx43a	0	s, d	-	s,d	1 (s,d,ax)	n.t.	n.t.	1 (d)	n.t.	n.t.	n.t.	1 (d,ax)	n.t.	n.t.
	tjx48a	n.t.	s, d	-	S,d	1 (d,ax)	n.t.	n.t.	1 (d)	n.t.	1 (s)	n.t.	1 (d,ax)	n.t.	n.t.
	tjx49a	n.t.	s, d	-	S,d	1 (d,ax)	n.t.	n.t.	1 (d)**	n.t.	n.t.	n.t.	1 (d,ax)	n.t.	n.t.
	tjx53a	n.t.	s, d	-	n.t.	1 (d,ax)	n.t.	n.t.	1 (d)	n.t.	n.t.	n.t.	1 (d)	n.t.	n.t.
	tjx55b	0	s, d	-	n.t.	1 (d,ax)	n.t.	n.t.	1 (d)	n.t.	n.t.	n.t.	1 (d,ax)	n.t.	n.t.
	tjx61a	0	s, d	-	n.t.	1 (d,ax)	n.t.	n.t.	1 (d)	n.t.	n.t.	n.t.	1 (d,ax)	n.t.	n.t.
	tjx69a	n.t.	s, d	-	n.t.	1 (d,ax)	n.t.	n.t.	1 (d)	n.t.	n.t.	n.t.	1 (d)	n.t.	n.t.
	tjx72b	n.t.	n.t.	-	n.t.	1 (s,d,ax)	n.t.	n.t.	1 (s,d)	n.t.	n.t.	n.t.	1 (s,d,ax)	n.t.	n.t.
	tjx72d	n.t.	n.t.	-	n.t.	1 (d,ax)	n.t.	n.t.	1 (d)	n.t.	n.t.	n.t.	1 (d,ax)	n.t.	n.t.
	tjx78a	n.t.	s, d	-	n.t.	1 (s)	n.t.	n.t.	1 (d)	n.t.	n.t.	n.t.	1 (d,ax)	n.t.	n.t.
	tjx86b	n.t.	s, d	-	n.t.	1 (d)	n.t.	n.t.	1 (d)	n.t.	n.t.	n.t.	1 (d)	n.t.	n.t.
	tjx87b	n.t.	D, s	-	n.t.	1 (d)	n.t.	n.t.	1 (d)	n.t.	n.t.	n.t.	1 (d)	n.t.	n.t.
CB+ dendrite-targeting	tjx21i	n.t.	d	-	n.t.	1 (s)	n.t.	n.t.	n.t.	n.t.	n.t.	n.t.	1 (s,d)**	0 (s,ax)	n.t.
	tjx22c	n.t.	d	-	D	1 (d)	0 (ax)	n.t.	0 (s,d)	n.t.	n.t.	0 (d)	1 (s)*	0 (s,ax)	1 (ax)
	tjx59b	n.t.	d	-	D	1 (d)	n.t.	n.t.	1 (s,d)	n.t.	n.t.	n.t.	n.t.	0 (s)	n.t.
AStria-projecting	tjx45a	n.t.	n.t.	n.t.	n.t.	1 (d)	0 (ax)	n.t.	1 (d)**	n.t.	n.t.	n.t.	1 (d,ax)	n.t.	1 (ax)
	tjx52a	n.t.	s,d*	s,d	S,d	0 (d)	n.t.	0 (d)	0 (d)	1 (ax)	n.t.	0 (s,d)	1 (d)	n.t.	1 (ax)
	tjx68a	n.t.	s,d*	s,d	s,D	0 (d)	n.t.	n.t.	0 (d)	n.t.	n.t.	n.t.	1 (d,ax**)	n.t.	n.t.
	tjx83c	n.t.	n.t.	n.t.	n.t.	1 (d)**	n.t.	n.t.	0 (d)	n.t.	n.t.	n.t.	1 (s,d,ax)**	n.t.	n.t.

Table S2. Postynaptic targets and neurochemical content of *in vivo*-recorded GABAergic neurons.
Postsynaptic targets: *: in lateral amygdala and AStria. 0 indicates the corresponding principal cell compartment was not in apposition with labeled axon varicosities. AIS: axon initial segment, ax: axon, d: dendrites, s: soma; capital letter: main targets. EM column: postsynaptic targets verified with electron microscopy.
Molecular markers: **: weakly positive, brackets: cell domain tested for immunoreactivity. 0 indicates the cell was concluded immunonegative, 1 indicates the cell was concluded immunopositive. CaMKIIα: Calcium/calmodulin dependent kinase II alpha subunit, CB1-R: cannabinoid receptor 1, DARPP-32: dopamine and cyclic adenosine 3',5'-monophosphate-regulated phosphoprotein with molecular weight of 32 kDa, GAD: glutamic acid decarboxylase, Nk1-R: neurokinin receptor 1, PV: parvalbumin, SOM: somatostatin, VGAT: vesicular GABA transporter.

Recorded neurons		Firing during dCA1 gamma nested in theta oscillations				Firing during dCA1 gamma, full recordings			
Cell type	Cell code	Mean angle	Modulation depth	p (Rayleigh)	n spikes	Mean angle	Modulation depth	p (Rayleigh)	n spikes
Axo-axonic	tjx20f	84.28	0.0253	0.878	202	88.75	0.0045	0.921	4130
	tjx27b	157.41	0.0405	0.218	929	251.54	0.0019	0.971	8377
	tjx56b	270.44	0.0244	0.412	1484	221.09	0.0100	0.430	8398
	tjx63a	222.25	0.0162	0.513	2532	213.39	0.0055	0.910	3099
	tjx66a	232.65	0.0269	0.102	3160	40.94	0.0055	0.951	1649
	tjx74a	68.78	0.0260	0.404	1345	223.63	0.0179	0.511	2094
PV+ basket	tjx38a	85.39	0.0253	0.117	3346	88.19	0.0078	0.800	3709
	tjx41c	153.20	0.0347	0.043	2613	205.47	0.0111	0.496	5688
	tjx42b	219.71	0.0063	0.901	2643	178.80	0.0238	0.096	4142
	tjx43a	235.37	0.0126	0.837	1120	141.69	0.0151	0.440	3626
	tjx48a	58.97	0.0050	0.887	4775	349.69	0.0266	0.043	4473
	tjx49a	328.40	0.0387	0.355	693	295.63	0.0104	0.537	5767
	tjx53a	253.44	0.0081	0.607	7684	237.82	0.0073	0.395	17507
	tjx55b	61.66	0.0068	0.958	913	28.85	0.0103	0.822	1848
	tjx61a	76.13	0.0096	0.713	3689	78.03	0.0087	0.358	13572
	tjx69a	113.46	0.0075	0.607	8911	144.17	0.0170	0.377	3393
	tjx72b	120.94	0.0251	0.239	2270	116.26	0.0073	0.573	10518
	tjx72d	24.18	0.0347	0.528	531	211.32	0.0172	0.111	7461
	tjx78a	215.22	0.0424	0.199	899	263.96	0.0091	0.435	10124
	tjx86b	210.76	0.0355	0.333	873	219.67	0.0060	0.601	13984
	tjx87b	298.55	0.0149	0.774	1151	80.85	0.0146	0.374	4584
CB+ dendrite-targeting	tjx21i	282.01	0.0021	0.995	996	106.73	0.0067	0.902	2285
	tjx22c	201.61	0.0080	0.934	1059	233.36	0.0065	0.790	5493
	tjx59b	60.46	0.0120	0.881	884	139.54	0.0177	0.135	6372
AStria-projecting	tjx45a	97.22	0.0224	0.401	1821	207.29	0.0086	0.754	3865
	tjx52a	91.77	0.0668	0.084	555	230.15	0.0287	0.044	3778
	tjx68a	0.59	0.0313	0.195	1674	27.63	0.0155	0.353	4346
	tjx83c	175.80	0.0211	0.611	1109	284.55	0.0129	0.340	6457

Table S3. Analysis of dCA1 gamma modulation of BLA interneuron’s spiking.

	Firing during dCA1 theta oscillations						Neurochemical content		
Cell code	Rate (Hz)	CV	Mean angle	Angular deviation	p (Rayleigh)	Modulation depth	CamKIIα	Calbindin	VGluT1
tjx23f	n.t.						1 (d)	1 (s)*	1 (ax)
tjx25e	0.63	1.25	n.s.	n.s.	7.7×10^{-1}	n.s.	n.t.	n.t.	1 (ax)
tjx47b	1.34	1.71	199.3	124.7	8.6×10^{-13}	0.09	1 (s)	1 (s)*	1 (ax)
tjx72c	0.50	2.26	186.9	93.0	2.4×10^{-26}	0.27	n.t.	n.t.	n.t.
tjx73a	0.06	2.43	n.s.	n.s.	8.2×10^{-1}	n.s.	1 (s)	1 (s)*	n.t.
tjx73b	0.06	2.25	323.7	98.7	2.0×10^{-10}	0.23	n.t.	n.t.	n.t.
tjx75a	0.44	2.00	n.s.	n.s.	9.0×10^{-1}	n.s.	1 (s)	1 (s)*	n.t.
tjx75b	0.33	1.22	302.0	103.0	3.1×10^{-11}	0.20	n.t.	n.t.	n.t.
tjx77b	0.19	2.22	n.s.	n.s.	7.9×10^{-2}	n.s.	1 (s)	0 (s)	1 (ax)
tjx79a	0.15	1.68	n.s.	n.s.	9.5×10^{-2}	n.s.	1 (s)	1 (s)*	1 (ax)
tjx79b	0.11	2.17	n.s.	n.s.	5.3×10^{-2}	n.s.	1 (s)	1 (s)*	1 (ax)
tjx81a	0.03	1.71	n.s.	n.s.	5.8×10^{-1}	n.s.	n.t.	1 (s)	1 (ax)
tjx81c	0.38	1.77	n.s.	n.s.	7.9×10^{-1}	n.s.	1 (s)	1 (s)*	1 (ax)
tjx82a	0.32	1.70	141.0	95.8	1.5×10^{-10}	0.25	1 (s)	1 (s)*	1 (ax)
tjx82b	0.29	2.52	n.s.	n.s.	3.9×10^{-1}	n.s.	1 (s)	0 (s)	1 (ax)
tjx83b	0.04	1.14	n.s.	n.s.	4.9×10^{-1}	n.s.	n.t.	n.t.	n.t.
tjx84b	0.19	1.95	n.s.	n.s.	4.7×10^{-1}	n.s.	1 (s)	1 (s)*	1 (ax)
tjx84c	0.03	1.34	n.s.	n.s.	3.1×10^{-1}	n.s.	1 (s)	1 (s)	1 (ax)
tjx86c	0.24	1.60	128.5	124.9	1.2×10^{-6}	0.09	1 (s)	0 (s)	1 (ax)
tjx88a	0.14	3.77	n.s.	n.s.	5.8×10^{-2}	n.s.	1 (s)	0 (s)	1 (ax)
tjx88b	0.13	2.73	n.s.	n.s.	1.9×10^{-1}	n.s.	1 (s)	0 (s)	n.t.
tjx89a	0.19	2.31	268.2	117.9	3.1×10^{-7}	0.12	1 (s)	0 (s)	1 (ax)
tjx89b	0.26	1.04	176.1	126.4	3.0×10^{-3}	0.09	n.t.	n.t.	n.t.
tjx89c	0.49	2.07	213.7	104.9	9.4×10^{-10}	0.19	1 (s)	1 (s)*	1 (ax)

Table S4. Principal cells: electrophysiological and immuno-cytochemical analysis.
0 indicates the cell was tested immunonegative, 1 indicates the cell was tested immunopositive. Brackets: cell domain tested for immunoreactivity. CaMKIIα: Calcium/calmodulin dependant kinase II alpha subunit, CV: coefficient of variation of firing, VGluT1: vesicular glutamate transporter 1. *: weakly immunopositive. ax: axon, d: dendrites, s: soma. n.s. not statistically significant, n.t. not tested.

Recorded neurons			Spike duration parameters (ms)				Spike amplitude parameters	
Cell type	Cell code	n spikes	Total width (c1-c4)	Baseline-trough (c1-c3)	1st peak-through (c2-c3)	Trough-2nd peak (c3-c5)	1st peak/through (a1/a2)	Amplitude (a1+a2, mV)
Axo-axonic	tjx20f	1412	1.06	0.57	0.33	0.86	1.13	2.16
	tjx27b	8630	1.08	0.55	0.33	0.83	1.35	1.55
	tjx56b	6519	1.09	0.55	0.32	0.76	1.76	0.98
	tjx63a	8231	1.00	0.49	0.28	0.65	1.60	1.39
	tjx66a	16475	0.85	0.48	0.28	0.68	0.97	0.80
	tjx74a	10241	1.29	0.58	0.36	0.98	2.03	2.44
	average	8585	1.06	0.54	0.32	0.79	1.47	1.55
	s.e.m.	2007	0.06	0.02	0.01	0.05	0.16	0.26
PV+ basket	tjx38a	10829	1.92	0.59	0.39	n.a.	1.72	0.87
	tjx41c	7774	0.79	0.48	0.27	0.58	0.84	0.98
	tjx42b	11985	1.17	0.46	0.26	n.a.	1.45	2.18
	tjx43a	2971	1.20	0.58	0.36	0.93	1.12	1.37
	tjx48a	15545	0.86	0.45	0.29	0.69	0.95	1.13
	tjx49a	6962	0.92	0.41	0.24	n.a.	2.19	1.65
	tjx53a	24510	1.16	0.43	0.26	n.a.	1.57	1.98
	tjx55b	5758	1.20	0.47	0.29	0.85	1.33	1.76
	tjx61a	2209	0.78	0.41	0.24	0.53	1.23	1.46
	tjx69a	25166	1.16	0.62	0.40	0.95	1.03	1.89
	tjx72b	4125	0.73	0.46	0.27	0.53	0.76	0.95
	tjx72d	4046	2.32	0.65	0.42	n.a.	1.75	2.21
	tjx78a	4051	1.49	0.60	0.38	1.17	1.54	4.62
	tjx86b	5713	2.29	0.73	0.49	n.a.	1.83	1.69
	tjx87b	5718	2.21	0.61	0.38	n.a.	2.47	2.15
	average	9157	1.35	0.53	0.33	0.78	1.45	1.79
	s.e.m.	1889	0.15	0.03	0.02	0.08	0.13	0.23
CB+ dendrite-targeting	tjx21i	15392	0.92	0.55	0.33	0.57	1.35	0.39
	tjx22c	3866	1.83	0.83	0.54	1.48	1.12	1.65
	tjx59b	2761	1.10	0.54	0.34	0.96	1.32	1.32
	average	7340	1.28	0.64	0.41	1.00	1.26	1.12
	s.e.m.	4039	0.28	0.10	0.07	0.26	0.07	0.38
AStria-projecting	tjx45a	5890	1.32	0.58	0.37	0.99	1.39	2.45
	tjx52a	7339	0.82	0.46	0.30	n.a.	2.94	1.07
	tjx68a	4722	1.28	0.58	0.39	n.a.	2.10	4.37
	tjx83c	3514	1.07	0.48	0.33	n.a.	1.90	2.47
	average	5366	1.12	0.52	0.35	0.99	2.08	2.59
	s.e.m.	817	0.11	0.03	0.02	n.a.	0.32	0.68
Principal neurons	tjx25e	601	1.59	0.74	0.46	1.21	1.37	0.97
	tjx47b	3785	1.79	0.96	0.65	1.52	1.26	1.56
	tjx72c	965	3.23	1.02	0.57	n.a.	2.04	4.97
	tjx73a	1045	2.50	0.95	0.63	n.a.	1.32	8.39
	tjx73b	1504	1.96	1.07	0.68	1.39	1.15	2.74
	tjx75a	732	2.21	1.18	0.79	1.63	1.35	3.98
	tjx75b	704	3.25	1.52	1.08	n.a.	1.77	2.38
	tjx77b	256	2.01	1.08	0.73	1.26	2.19	2.15
	tjx79a	619	2.92	1.12	0.81	n.a.	1.85	2.88
	tjx79b	528	2.61	1.16	0.85	1.84	1.69	3.51
	tjx81a	199	2.04	1.07	0.73	1.66	1.98	2.15
	tjx81c	1246	1.91	0.98	0.65	1.48	1.67	2.24
	tjx82a	280	2.27	1.17	0.81	1.81	1.60	1.89
	tjx82b	2001	2.18	1.09	0.68	1.63	1.57	1.55
	tjx83b	325	1.64	0.97	0.63	1.09	1.39	2.19
	tjx84b	768	2.64	1.00	0.72	n.a.	2.75	3.51
	tjx84c	196	2.72	1.20	0.85	1.93	1.64	2.72
	tjx86c	2033	1.51	0.90	0.62	0.93	1.80	1.98
	tjx88a	1860	1.59	0.95	0.63	1.19	1.48	3.21
	tjx88b	334	2.86	1.36	0.98	0.00	1.84	2.68
	tjx89a	1214	1.53	0.89	0.60	1.09	1.75	1.25
	tjx89b	641	1.87	0.97	0.62	1.49	1.51	1.38
	tjx89c	2172	1.85	0.98	0.68	1.26	1.98	3.28
	average	1044	2.20	1.06	0.71	1.36	1.69	2.76
	s.e.m.	180	0.11	0.03	0.03	0.10	0.07	0.32

Table S5. Spike waveform parameters of all GABAergic and principal neurons.
All spike duration parameters were smaller for interneurons ($p < 0.0001$, Mann-Whitney U-test), except trough-2nd peak duration,. The 1st peak/trough amplitude ratio did not differ between interneurons and principal cells ($p > 0.1$, Mann-Whitney U-test); See Figure S8A for cursor names (c1 to c5, a1 and a2).
n.a.: not applicable.

BLA recording	Ventral HPC reference					Dorsal CA1 reference		
Cell code	Electrode location	Distance to str. pyramidale (μm)	Preferred theta phase (degrees)	Modulation depth	p (Rayleigh test)	Preferred theta phase (degrees)	Modulation depth	p (Rayleigh test)
tx87b (PV+ basket)	vCA1 (str. radiatum)	355.00	62.2	0.03	1.1×10^{-2}	183.5	0.04	5.7×10^{-9}
tjx88a (principal cell)	vSubiculum (str. radiatum)	450.00	329.3	0.06	4.9×10^{-2}	n.s.	n.s.	5.8×10^{-2}
tjx88b (principal cell)	vSubiculum (str. radiatum)	450.00	n.s.	n.s.	2.1×10^{-1}	n.s.	n.s.	1.9×10^{-1}
tjx89a (principal cell)	vSubiculum (str. radiatum)	360.00	196.5	0.13	4.6×10^{-7}	268.2	0.12	3.1×10^{-7}
tjx89b (principal cell)	vSubiculum (str. radiatum)	360.00	124.4	0.07	4.1×10^{-2}	176.1	0.09	3.0×10^{-3}
tjx89c (principal cell)	vSubiculum (str. radiatum)	360.00	117.3	0.17	2.9×10^{-7}	213.7	0.19	9.4×10^{-10}

Recording	Phase differences (degrees)	
Cell code	Neuron phase difference (dCA1 - vCA1)	LFP phase difference
tx87b	121.3	101.2
tjx88a	n.a.	32.9
tjx88b	n.a.	39.0
tjx89a	71.6	59.8
tjx89b	51.7	55.7
tjx89c	96.45	78.0

Table S6. Theta phase modulation of BLA neuron firing assessed with dorsal and ventral hippocampal references.
n.a.: not applicable; n.s.: not statistically significant.

Molecule	Species	Provider	Code / Catalogue number	Dilution	Specificity
Ankyrin G	Mouse	UC Davis/NIH NeuroMab Facility	75-146 (clone N106/36)	1:2,000-1:4,000	Manufacturer informations and labels axon initial segments and Ranvier nodes, as published with other antibodies.
Calbindin	Mouse	Swant	300	1:250-400	(Celio, 1990)
	Rabbit	Swant	CB38	1:5,000	(Airaksinen et al., 1997) and manufacturer informations.
CaMKIIα	Mouse	Abcam	ab22609 (clone 6G9)	1:500	(Erondu and Kennedy, 1985)
Calretinin	Goat	Swant	CG1	1:1,000-2,000	Manufacturer informations and labeling pattern as published with other antibodies.
CB1-R	Guinea Pig	Dr. M. Watanabe, Hokkaido University	-	314 $ng.mL^{-1}$	(Fukudome et al., 2004)
DARPP-32	Mouse	Prof. Drs. P. Greengard and A. Nairn	Clone D32 6a	1:250	(Hemmings and Greengard, 1986; Ouimet et al., 1984)
	Rabbit	Cell Signaling Technology	2302	1:200	(Partida et al., 2004)
$GABA_A$-Rα1	Rabbit	Prof. Dr. J.M. Fritschy, University of Zurich	-	1:10,000	(Benke et al., 1991)
GAD	Mouse	Chemicon/Millipore	MAB351R, (clone GAD-6)	1:500	(Chang and Gottlieb, 1988)
Kv 3.2	Rabbit	Alomone	APC-011	1:500	(McDonald and Mascagni, 2006) and manufacturer information.
Nk1-R	Guinea pig	Chemicon/Millipore	AB5800	1:700	Manufacturer information and labeling pattern as published with other antibodies.
Parvalbumin	Guinea pig	Synaptic systems	195-004	1:2,500-5000	Labeling pattern as published with other antibodies.
	Goat	Swant	PVG-214	1:2,000	Manufacturer information and labeling pattern as published with other antibodies.
Somatostatin	Mouse	GeneTex	GTX7 1935 (clone SOM-018)	1:250	Manufacturer information and labeling pattern as published with other antibodies.
VGAT	Rabbit	Synaptic systems	131-003	1:500	Manufacturer information and labeling pattern as published with other antibodies.
VGluT1	Mouse	Synaptic systems	135-311	1:500	Manufacturer information and labeling pattern as published with other antibodies.

Table S7. Primary antibodies used.

CaMKIIα: Calcium/calmodulin dependant kinase II alpha subunit, CB1-R: cannabinoid receptor 1, DARPP-32: dopamine and cyclic adenosine 3', 5'-monophosphate-regulated phosphoprotein with molecular weight of 32 kDa, GAD: glutamic acid decarboxylase, Nk1-R: neurokinin receptor 1, VGAT: vesicular GABA transporter, VGluT1: vesicular glutamate transporter 1.

SUPPLEMENTAL EXPERIMENTAL PROCEDURES

Principal cells: recordings and analysis

Recordings were obtained as described for GABAergic cells in the main text, from 15 of the 70 rats used in the study. Recordings lasted 75 ± 42 min (mean ± s.d., range 19-183 min) and yielded 175-3,172 spikes during dCA1 theta oscillations. Owed to the firing activities of principal neurons, their firing rates and coefficients of variation were calculated over time periods containing 50 spikes (same periods for firing rate and CV). Cells were confirmed as glutamatergic principal neurons if they expressed the vesicular glutamate transporter 1 (VGluT1) in axon varicosities. We also tested immunoreactivity for CaMKIIα and calbindin.

Anatomically-identified principal cells fired wide spikes at low rates, with frequent spike bursts. Five putative (unlabeled) principal neurons were included for analysis based on this clear electrophysiological signature: broad spikes, low firing rates and occurrence of spike bursts. Their firing patterns and spike features were not different from that of identified neurons (see Table S4 and Figure S8).

The mean firing rate of all BLA principal cells studied here was 0.29 Hz, which is higher than that reported in an unbiased study of BLA projection cells' firing (0.09 Hz) (Gaudreau and Pare, 1996), but lower than reported using techniques based on spike sorting of spontaneously active units (~2 Hz) (Herry et al., 2008) . By slowly (~1 $\mu m.s^{-1}$) advancing the recording electrode, we did not bias our recordings towards the most active principal cells. However, only spontaneously firing (but not silent cells) are recorded extracellularly. In addition, our phase analysis required at least 100 spikes to have occurred during hippocampal theta periods. Cells silent during these epochs were therefore excluded.

Only one principal cell in our sample fired at a rate above 1 Hz, indicating that neurons with spontaneous firing rate > 1 Hz are almost exclusively GABAergic interneurons. Moreover, all the neurons labeled when our recordings were biased towards units with large action potentials, burst firing and low firing frequencies were confirmed to be glutamatergic neurons. These data suggest that firing rates and spike features (see above) can be used in combination to reliably distinguish interneurons from principal neurons recorded extracellularly.

All confirmed BLA glutamatergic neurons (expressing VGluT1) also expressed CaMKIIα (n= 14/14 tested, Table S4). In the BLA, CaMKIIα thus appears to be a specific (McDonald et al., 2002) and sensitive (the present result) marker of glutamatergic principal neurons. This validates our method of interneurons' postsynaptic target identification at light and electron microscopy levels. Calbindin (CB) is expressed in a subset of BLA principal neuron ((Kemppainen and Pitkanen, 2000; McDonald, 1997), 12/18 here). Of the electrophysiological parameters we studied (Table S4), only CV differed between CB+ and CB- neurons. Calbindin-negative principal neurons had higher CVs (CB- vs. CB+, mean 2.5 vs. 1.9, unpaired t-test; p= 0.016), likely indicating that they generated more spike bursts. This result is consistent with stronger bursting activity in deep CA1 pyramidal cells ((Mizuseki et al., 2011), most of which are CB-) compared with superficial cells (most of which are CB+ (Baimbridge and Miller, 1982)).

Single principal cells electrophysiological and immunocytochemical data are summarized in Table S4.

Ventral hippocampal recordings and analysis

In 3 experiments, ventral hippocampal LFPs (vCA1-subiculum) were recorded with a glass electrode simultaneously with BLA, dCA1 and ECoG signals (recorded as stated in Experimental Procedures).

To reach the vCA1-subiculum, a medio-lateral 20˚ angle was used. Since theta phase gradually shifts in str. radiatum (Lubenov and Siapas, 2009), we initially aimed to record in str. pyramidale. The position of vCA1-subiculum str. pyramidale was identified online by the recording of several units within a short electrode trajectory (~100 μm), which fired long-duration spikes and complex spike bursts. We found low amplitude, intermittent theta oscillations in str. pyramidale of vCA1-subiculum (Figure S9A). In str. radiatum, theta oscillations were transient, but more frequent and of higher amplitude than those in str. pyramidale (Figure S9B). These differences are reflected in the corresponding power spectra (Figure S9C). Therefore, str. radiatum LFP signal was used as a second reference.

At the end of the experiment, a Neurobiotin deposit was made (see Experimental Procedures) in str. pyramidale. Str. radiatum recording locations (as shown in Figure S9D and Figure 7E) were extrapolated from the electrode trajectories and the documented distances to the Neurobiotin deposits in str. pyramidale.

Power spectra as illustrated in Figure S9C were obtained as follows. After applying a 0.5 Hz high pass filter, fast Fourier transforms of LFPs were computed over 200 s of robust dCA1 theta oscillations. Data were windowed with a Hanning filter (0.1995 Hz windows) and power spectra were constructed (Spike2).

Neuron firing relations to ventral hippocampal theta were computed from the same time periods as for dCA1 theta analysis. This allowed for the comparison between preferred phases computed with the two references. Statistical significance threshold of Rayleigh's test was reduced to 0.05 for analysis of BLA neuron modulation by ventral hippocampal theta.

Brains fixation and sectioning

The animals were given a lethal dose of ketamine 30 min to 4 h after juxtacellular labeling, and perfused via the ascending aorta with ~60 mL of Phosphate Buffer Saline (PBS, pH 7.4), followed by 300 mL of fixative containing 4% w/v paraformaldehyde, 0.1% v/v glutaraldehyde, 15% v/v saturated picric acid, in PB 0.1 M, pH~7.3 (100 mL at 20 $mL.min^{-1}$ then 200 mL at 10 $mL.min^{-1}$). Brains were dissected out and stored overnight in PBS. They were cut in 60 µm-thick coronal sections with a vibrating microtome (Leica VT-1000), extensively washed in PBS, and conserved in PB 0.1 M with 0.05% NaN_3 until use.

Immunofluorescence: Neurobiotin-filled neurons were visualized with streptavidin conjugated to a fluorophore, then tested for their neurochemical content using immunofluorescence. All reagents were diluted in PBS containing 0.15% v/v triton X-100. Free-floating sections were blocked in 20% normal horse serum (NHS, Vector laboratories) for 1h at room temperature and incubated at 4˚C for 2 days in one or several primary antibodies with 2% NHS (antibodies listed in Table S7, with references showing their specificity). Sections were washed, and incubated at 4˚C overnight with appropriate highly cross-adsorbed secondary antibodies, coupled to Cy3, Cy5, Dylight 405 (Jackson ImmunoResearch Laboratories) or Alexa 488 (Invitrogen), with 2% NHS. After several washes, sections were mounted in Vectashield (Vector Laboratories).

Immunoreactivity was evaluated using epifluorescence (Leica DMRB microscope fitted with a 40x 0.7 NA objective), structured illumination (Apotome system operated with Axiovision software) and confocal microscopy (LSM 710

operated with Zen 2008 software) with a AxioImager Z1 microscope fitted with 40x NA 1.3 and 63x NA 1.4 oil objectives (hardware and software from Carl Zeiss Ltd.). For confocal imaging, single-channel sequential frame scanning was used to avoid crosstalk. Pinhole size was adjusted to preserve a constant optical slice thickness across channels (typically 1.1 μm for 40x, 0.7 μm for 63x). Beam splitter and wavelength acquisition bands were adjusted using the "Smart" function of Zen software. Wavelength acquisition bands were occasionally narrowed to avoid acquiring signal resulting from excitation crosstalk. Optimal pixel size was set using the built-in function of Zen and kept constant for all channels.

Absence of crosstalk was ensured by absence of "copy" of one channel in another in the section analyzed and in a single-stained control sample. A neuron was concluded immunopositive if the staining pattern was as expected, crosstalk excluded and co-localization evident with the relevant compartments of the streptavidin-labeled neuron. If no colocalization was observed, the neuron was concluded negative. If the staining was suboptimal or crosstalk persisted, the cell was listed as not tested. Brightness and contrast were adjusted for entire image frames using Photoshop (Adobe CS3).

Preferred postsynaptic targets (putative) were determined qualitatively with fluorescence and/or transmission light microscopy. A majority of axon varicosities making close appositions with CaMKIIα+ somata and large calibre dendrites in the BLA indicated the neuron was a basket cell. A majority of appositions with ankyrin G+ axon initial segments (AIS) defined the cell as axo-axonic (Gulyas et al., 2010). When the axon made apposition with CaMKIIα+ dendrites and with non-apparent structures (potentially too small for detection with light microscopy), the cell was classified as dendrite-targeting.

Electron microscopy was used to validate light microscopic observations and to evaluate quantitatively postsynaptic target preference. Indeed, light microscopy gives an estimate of preferred targets but can be inaccurate in quantifying their relative proportions (Tamas et al., 1997).

Sections were cryoprotected with 20% w/v sucrose in PB 0.1 M and freeze–thawed over liquid nitrogen to enhance penetration of reagents. Sections were then incubated with a streptavidin-biotinylated horseradish peroxidase (HRP) complex (ABC Elite kit, Vector Laboratories) and Neurobiotin-filled cells were visualised with

HRP reaction products using nickel-intensified diaminobenzidine (DAB) as a chromogen. Postsynaptic profiles belonging to principal neurons were identified at electron microscopic level with an immunoperoxidase method against CaMKIIα. Sections were blocked in 20% normal goat serum (NGS, Vector laboratories) for 1h at room temperature, incubated at 4˚C for 2 days in 1:500 mouse anti-CaMKIIα (Table S7) in PB 0.1 M NGS 2%. Sections were washed and incubated overnight at 4˚C with a HRP-coupled goat anti-mouse antibody (Dako) diluted 1:100 in PB 0.1 M with NGS 2%. Sections were washed and reacted with non-intensified DAB.

Sections were postfixed with 1% OsO4 (TAAB Laboratory Equipment; in PB 0.1 M), block-stained in 1% uranyl acetate (TAAB), dehydrated, embedded in epoxy resin (Durcupan; Fluka) and mounted on glass slides. Axon-rich areas were cut out and re-embedded in resin. Serial ultrathin sections (70 nm) were cut with an ultramicrotome (Ultracut S; Leica) and collected on Pioloform-coated copper slot grids.

Analysis of the specimens was performed using a Philips CM 120 electron microscope. Axons were examined for the identity of postsynaptic targets without further contrasting. Sampling randomization was achieved as follows: blocks were cut out from random axon-rich areas and all axon profiles were followed and analyzed until 20 synapses were collected. In some instances, our analysis yielded additional synapses, which were also included for quantification. Postsynaptic targets were always followed in serial sections. Their identity was determined using standard criteria (Peters et al., 1991). When the target was a dendrite, its diameter (defined as its smaller axis) was measured. In sections where CaMKIIα was stained with DAB, clear DAB-labeled structures were considered as part of glutamatergic neurons. Because in our samples many small dendrites and dendritic spines were not labeled, we did not conclude on the identity of structures in which HRP product was not detected.

Camera lucida reconstructions of neurons were performed using a drawing tube mounted onto a Zeiss Axioplan2 microscope with a 63x oil 1.4 NA objective. Amygdala nuclei boundaries were drawn from one of the 2 sections from which the axon was reconstructed. We used adjacent coronal sections and, in several instances immunohistochemistry, to determine boundaries between lateral and basal amygdala nuclei.

Modulation of BLA interneuron's firing by CA1 gamma oscillations

Neuronal spiking modulation with dCA1 gamma oscillation was assessed according to the method published in Tukker et al. (2007), using the same custom Spike2 analysis program.

LFPs recorded in dCA1 were digitally low pass-filtered (300 Hz) off-line. LFPs were then down-sampled to 1 kHz and digitally filtered to extract gamma oscillations (bandpass 30–80 Hz Finite Impulse Response filter). For firing modulation analysis, we selected the gamma cycles with amplitude greater than the mean amplitude calculated over the entire duration of the recording. Two types of gamma oscillation epochs were analyzed: those occurring specifically during theta oscillations (epochs as defined in Experimental Procedures), and those occurring indifferently throughout the recording.

Gamma oscillation troughs were detected and gamma cycle angles were linearly interpolated between consecutive troughs. Rayleigh's test was used to assess modulation of individual neurons in phase with dCA1 gamma (significance threshold p= 0.005; more details on Rayleigh's test can be found in Experimental Procedures).

Extracellular spike waveform analysis

For all interneurons (n= 28) and principal cells (n= 23), extracellular spikes detected during theta oscillations (see Experimental Procedures for theta epochs detection) were averaged from the BLA "unit" channel (300-5000 Hz filter). The event-triggered averaging function of Spike 2 was applied offline, using spike time stamps as a trigger.

On these averaged spike waveforms, reference cursors were placed as follows: c1 at the start of the first positive deflection, c2 at the first peak, c3 at the trough, c4 when the spike reached baseline again, c5 at the second peak (Figure S8A). Five parameters were measured and compared across cell classes. Four spike duration values: total width (c1-c4), baseline to trough (c1-c3), first peak to trough (c2-c3), trough to second peak duration (c3-c5); one amplitude parameter: the 1st peak / trough amplitude ratio. In some cells, it was not possible to determine the last parameter of spike duration due to the lack of second peak. In addition to these, spike amplitude was measured from 1st positive peak to trough.

Kruskal Wallis test was used to compare values across interneuron types. Mann-Whitney U-test was used to compare values measured in interneurons versus principal cells. Unsupervised hierarchical cluster analysis was performed to further investigate

whether cell types could be separated on the basis of their spike waveforms. Two parameters were included: the 1st peak to trough duration (c2-c3) and the 1st peak/trough amplitude ratio (a1/a2). Clustering was performed using the Euclidean distance followed by the minimum variance ward algorithm embedded in Matlab (MathWorks). The lowest (> 1) number of final clusters was determined as the one that provided a robust inconsistency coefficient value.

SUPPLEMENTAL REFERENCES

Airaksinen, M.S., Eilers, J., Garaschuk, O., Thoenen, H., Konnerth, A., and Meyer, M. (1997). Ataxia and altered dendritic calcium signaling in mice carrying a targeted null mutation of the calbindin D28k gene. Proc Natl Acad Sci U S A *94*, 1488-1493.

Baimbridge, K.G., and Miller, J.J. (1982). Immunohistochemical localization of calcium-binding protein in the cerebellum, hippocampal formation and olfactory bulb of the rat. Brain Res *245*, 223-229.

Benke, D., Cicin-Sain, A., Mertens, S., and Mohler, H. (1991). Immunochemical identification of the alpha 1- and alpha 3-subunits of the GABAA-receptor in rat brain. J Recept Res *11*, 407-424.

Celio, M.R. (1990). Calbindin D-28k and parvalbumin in the rat nervous system. Neuroscience *35*, 375-475.

Chang, Y.C., and Gottlieb, D.I. (1988). Characterization of the proteins purified with monoclonal antibodies to glutamic acid decarboxylase. J Neurosci *8*, 2123-2130.

Erondu, N.E., and Kennedy, M.B. (1985). Regional distribution of type II Ca2+/calmodulin-dependent protein kinase in rat brain. J Neurosci *5*, 3270-3277.

Fukudome, Y., Ohno-Shosaku, T., Matsui, M., Omori, Y., Fukaya, M., Tsubokawa, H., Taketo, M.M., Watanabe, M., Manabe, T., and Kano, M. (2004). Two distinct classes of muscarinic action on hippocampal inhibitory synapses: M2-mediated direct suppression and M1/M3-mediated indirect suppression through endocannabinoid signalling. Eur J Neurosci *19*, 2682-2692.

Gaudreau, H., and Pare, D. (1996). Projection neurons of the lateral amygdaloid nucleus are virtually silent throughout the sleep--waking cycle. J Neurophysiol *75*, 1301-1305.

Gulyas, A.I., Szabo, G.G., Ulbert, I., Holderith, N., Monyer, H., Erdelyi, F., Szabo, G., Freund, T.F., and Hajos, N. (2010). Parvalbumin-containing fast-spiking basket cells generate the field potential oscillations induced by cholinergic receptor activation in the hippocampus. J Neurosci *30*, 15134-15145.

Hemmings, H.C., Jr., and Greengard, P. (1986). DARPP-32, a dopamine- and adenosine 3':5'-monophosphate-regulated phosphoprotein: regional, tissue, and phylogenetic distribution. J Neurosci *6*, 1469-1481.

Herry, C., Ciocchi, S., Senn, V., Demmou, L., Muller, C., and Luthi, A. (2008). Switching on and off fear by distinct neuronal circuits. Nature *454*, 600-606.

Kemppainen, S., and Pitkanen, A. (2000). Distribution of parvalbumin, calretinin, and calbindin-D(28k) immunoreactivity in the rat amygdaloid complex and colocalization with gamma-aminobutyric acid. J. Comp. Neurol. *426*, 441-467.

Likhtik, E., Pelletier, J.G., Popescu, A.T., and Pare, D. (2006). Identification of basolateral amygdala projection cells and interneurons using extracellular recordings. J Neurophysiol *96*, 3257-3265.

Lubenov, E.V., and Siapas, A.G. (2009). Hippocampal theta oscillations are travelling waves. Nature *459*, 534-539.

Mallet, N., Pogosyan, A., Marton, L.F., Bolam, J.P., Brown, P., and Magill, P.J. (2008). Parkinsonian beta oscillations in the external globus pallidus and their relationship with subthalamic nucleus activity. J Neurosci *28*, 14245-14258.

McDonald, A.J. (1997). Calbindin-D28k immunoreactivity in the rat amygdala. J. Comp. Neurol. *383*, 231-244.

McDonald, A.J., and Betette, R.L. (2001). Parvalbumin-containing neurons in the rat basolateral amygdala: morphology and co-localization of Calbindin-D(28k). Neuroscience *102*, 413-425.

McDonald, A.J., and Mascagni, F. (2006). Differential expression of Kv3.1b and Kv3.2 potassium channel subunits in interneurons of the basolateral amygdala. Neuroscience *138*, 537-547.

McDonald, A.J., Muller, J.F., and Mascagni, F. (2002). GABAergic innervation of alpha type II calcium/calmodulin-dependent protein kinase immunoreactive pyramidal neurons in the rat basolateral amygdala. J Comp Neurol *446*, 199-218.

Mizuseki, K., Diba, K., Pastalkova, E., and Buzsaki, G. (2011). Hippocampal CA1 pyramidal cells form functionally distinct sublayers. Nat Neurosci *14*, 1174-1181.

Ouimet, C.C., Miller, P.E., Hemmings, H.C., Jr., Walaas, S.I., and Greengard, P. (1984). DARPP-32, a dopamine- and adenosine 3':5'-monophosphate-regulated phosphoprotein enriched in dopamine-innervated brain regions. III. Immunocytochemical localization. J Neurosci *4*, 111-124.

Partida, G.J., Lee, S.C., Haft-Candell, L., Nichols, G.S., and Ishida, A.T. (2004). DARPP-32-like immunoreactivity in AII amacrine cells of rat retina. J Comp Neurol *480*, 251-263.

Paxinos, G., and Watson, C. (2007). The Rat Brain in Stereotaxic Coordinates, 6th edition (Elsevier).

Peters, A., Palay, S.L. & Webster, H. deF. (1991). The fine structure of the nervous system, Oxford University Press.

Tamas, G., Buhl, E.H., and Somogyi, P. (1997). Fast IPSPs elicited via multiple synaptic release sites by different types of GABAergic neurone in the cat visual cortex. J Physiol *500 (Pt 3)*, 715-738.

Tukker, J.J., Fuentealba, P., Hartwich, K., Somogyi, P., and Klausberger, T. (2007). Cell type-specific tuning of hippocampal interneuron firing during gamma oscillations in vivo. J Neurosci *27*, 8184-8189.
